# Identification of bolting-related microRNAs and their targets reveals complex miRNA-mediated flowering-time regulatory networks in radish (*Raphanus sativus* L.)

**DOI:** 10.1038/srep14034

**Published:** 2015-09-15

**Authors:** Shanshan Nie, Liang Xu, Yan Wang, Danqiong Huang, Everlyne M. Muleke, Xiaochuan Sun, Ronghua Wang, Yang Xie, Yiqin Gong, Liwang Liu

**Affiliations:** 1 National Key Laboratory of Crop Genetics and Germplasm Enhancement; Key Laboratory of Biology and Genetic Improvement of Horticultural Crops (East China), Ministry of Agriculture of P.R.China; College of Horticulture, Nanjing Agricultural University, Nanjing 210095, P.R. China; 2 Department of Plant Sciences, North Dakota State University, Fargo, ND 58108, USA

## Abstract

MicroRNAs (miRNAs) play vital regulatory roles in plant growth and development. The phase transition from vegetative growth to flowering is crucial in the life cycle of plants. To date, miRNA-mediated flowering regulatory networks remain largely unexplored in radish. In this study, two small RNA libraries from radish leaves at vegetative and reproductive stages were constructed and sequenced by Solexa sequencing. A total of 94 known miRNAs representing 21 conserved and 13 non-conserved miRNA families, and 44 potential novel miRNAs, were identified from the two libraries. In addition, 42 known and 17 novel miRNAs were significantly differentially expressed and identified as bolting-related miRNAs. RT-qPCR analysis revealed that some miRNAs exhibited tissue- or developmental stage-specific expression patterns. Moreover, 154 target transcripts were identified for 50 bolting-related miRNAs, which were predominately involved in plant development, signal transduction and transcriptional regulation. Based on the characterization of bolting-related miRNAs and their target genes, a putative schematic model of miRNA-mediated bolting and flowering regulatory network was proposed. These results could provide insights into bolting and flowering regulatory networks in radish, and facilitate dissecting the molecular mechanisms underlying bolting and flowering time regulation in vegetable crops.

The transition from vegetative to reproductive growth is a crucial event in the life cycle of plants. An intricate genetic circuitry has evolved to control the onset of flowering in response to diverse endogenous pathways and exogenous stimuli^1^. In plants, the complexity of regulation ensures that bolting and flowering is initiated at a condition that is suitable and favorable for successful reproduction. The molecular and genetic analysis in *Arabidopsis thaliana* has revealed that there are five major interactive pathways involved in flowering control including photoperiod pathway, vernalization pathway, autonomous pathway, gibberellin (GA) pathway and age pathway^1^,^2^,^3^. During the past two decades, a set of genes associated with the transition to flowering have been identified and integrated into these cross-regulating pathways^1^,^4^. Although the biological functions of some key flowering genes have been extensively characterized in many plant species^4^, the molecular mechanisms of flowering-time regulation are still not fully understood in some root vegetable crops including radish (*Raphanus sativus* L.).

MicroRNAs (miRNAs) are a class of endogenous non-coding small RNAs with approximately 21 to 24 nucleotides (nt) in animals and plants^5^. In plants, miRNAs are processed from long primary transcripts cleaved by Dicer-like 1 RNase (DCL1) and subsequently incorporated into the RNA-induced silencing complex (RISC), where miRNAs are complementary to specific target mRNAs and regulate their expressions post-transcriptionally by guiding the cleavage of complementary mRNAs^6^,^7^. Many plant miRNAs are evolutionally conserved among many species^8^,^9^. Recent evidences have indicated that miRNAs could play pivotal roles in various plant growth and developmental processes, such as hormone homeostasis^10^,^11^, root development^12^,^13^, leaf morphogenesis^14^,^15^, flower development^11^,^16^,^17^, embryogenesis^18^,^19^ and stress responses^20^,^21^.

Recently, an increasing number of studies have revealed miRNAs as master regulators of gene expressions to be implicated in the developmental transition from vegetative growth to flowering^4^,^8^,^22^. With the development of high-throughput sequencing (next-generation sequencing, NGS) technology, a large number of miRNAs related to flowering and flower development have been identified and characterized in several important species, such as trifoliate orange^23^, rice^24^, hickory^17^, *Xanthoceras sorbifolia*^25^ and poplar^26^. Several miRNAs, such as miR156 and miR172, two major players in flowering regulation, have been shown to regulate the floral transition and are also integrated into flowering pathways^22^,^27^. Age pathway is a newly defined endogenous flowering pathway and mediated by miR156, which targets *Squamosa Promoter Binding Protein Like* (*SPL*) transcription factors and decreases with increasing age of the plant^2^,^3^. Overexpression of miR156 could delay flowering and prolong vegetative stage in many species including *Arabidopsis*^28^, rice^29^ and tomato^30^. The miR172, targeting *APETALA2* (*AP2*)*-like* transcription factors, has the opposite effect to the miR156 on the regulation of flowering time and increases with phase development in *Arabidopsis*^31^,^32^, which could induce floral organogenesis and promote flowering^32^,^33^. In addition, some miRNAs including miR159^34^,^35^, miR167^16^, miR169^36^, miR393^37^ and miR399^38^, were found to be involved in regulation of flowering time. For instance, GA pathway is governed by miR159, which represses the regulation of MYB transcription factors^4^,^22^,^34^. The overexpression of miR159 could delay flowering and its roles in manipulating flowering time control have been clarified in *Arabidopsis*^34^ and *Sinningia speciosa*^35^. These studies showed that miRNAs play important roles in a number of developmental processes and pathways which control flowering time. However, there is little information about miRNA-mediated gene expressions and flowering-time regulatory networks, especially in vegetable crops.

Radish (2*n* = 2*x* = 18), belonging to the Brassicaceae family, is one of the most economically important root vegetable crops worldwide. In the entire life cycle of radish, bolting and flowering are integrated steps. Premature bolting is a destructive problem in radish production as it causes significant reduction in yield and quality of the economic product in Brassicaceae crops. The optimum timing of bolting and flowering is vital for economy organ growth in preventing radish bolting and flowering prematurely. Therefore, elucidating the miRNA-directed genetic networks of radish bolting- and flowering-time modulation is imperative. Although using high-throughput sequencing, some tissue-specific as well as heavy metal-responsive miRNAs have been extensively identified in radish^19^,^20^,^21^,^39^. Nevertheless, there are no reports on systematic identification and characterization of bolting-related miRNAs in radish. To explore the roles of miRNAs in bolting- and flowering-time regulation, two small RNA libraries (NAU-VS and NAU-RS) from radish leaves at vegetative stage and reproductive stage were constructed and sequenced using Solexa sequencing. The objectives of this study were to identify known and potential novel miRNAs from radish leaves and to validate target genes of bolting- and flowering-related miRNAs. These results revealed complex miRNA-mediated flowering-time regulatory network in radish, and provided insights into clarification of the molecular mechanisms underlying the regulation of plant bolting and flowering.

## Results

### High-throughput sequencing of transcriptome and small RNAs in radish leaf

To obtain a comprehensive overview of the radish leaf transcriptome, an mRNA library from equally mixed radish leaves at vegetative and reproductive stages was sequenced using Solexa sequencing, with the generation of 58.6 million raw reads. After removing poly (A) tails, low-quality tags and adaptor contaminants, a total of 54.6 million clean reads were obtained. Then all high-quality clean reads were assembled into 111,167 contigs. After further pair-end annotation and gap filling, these contigs were assembled into 53,642 unigenes, with an average length of 802 nt and a N50 length of 1035 nt. Our radish leaf transcriptome library integrated with the available genomic survey sequences (GSS) and expressed sequence tag (EST) sequences were used as the radish reference sequences for the identification of known and novel miRNAs in radish.

**Figure 1:**
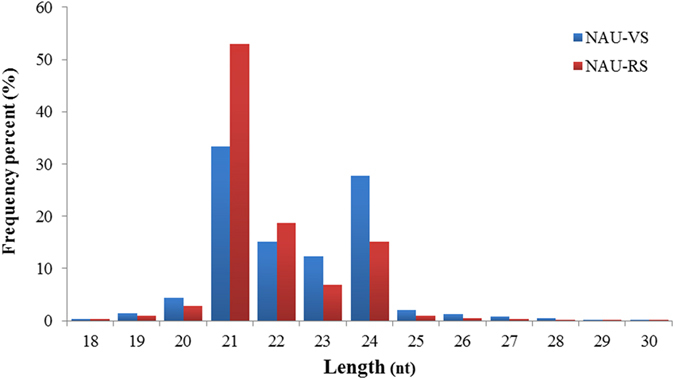
Length distribution and frequency percent of small RNA sequences in NAU-VS and NAU-RS libraries in radish.

To identify miRNAs during radish bolting and flowering, two sRNA libraries, ‘NAU-VS’ and ‘NAU-RS’, were constructed from radish leaves at vegetative and reproductive stages, respectively. In total, 36,841,873 raw reads representing 7,497,083 unique sequences were obtained from the two sRNA libraries (Table 1; Supplementary Fig. S1). After filtering out the low quality reads, contaminants and adapter sequences, 18,948,210 (representing 4,992,584 unique sequences) and 17,893,663 (representing 3,491,761 unique sequences) clean reads were obtained from NAU-VS and NAU-RS libraries, respectively (Table 1). The length of total sRNA ranged from 18 to 30 nt, with 21-nt sRNAs being the most abundant in both libraries, which accounted for 33.33% and 52.97% in NAU-VS and NAU-RS libraries, respectively (Fig. 1). By comparing with the NCBI GenBank and Rfam databases, these sRNA sequences that matched non-coding sRNAs included tRNAs, rRNAs, snoRNAs and snRNAs were removed (Supplementary Table S2). Thereafter, 61,574 (NAU-VS) and 50,222 (NAU-RS) miRNA analogous sequences were annotated and obtained for known miRNA identification. The remaining 4,846,072 (NAU-VS) and 3,375,354 (NAU-RS) unannotated sRNAs were used to predict potentially novel miRNAs for subsequent analysis.

### Identification of known miRNAs in radish leaf

**Figure 2:**
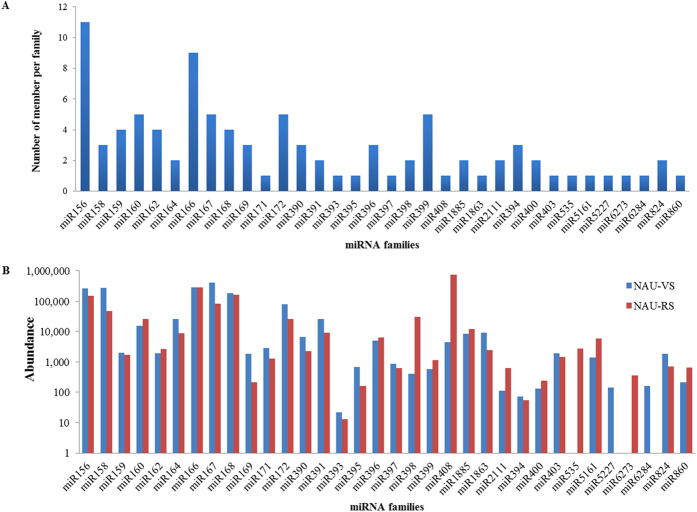
Members and abundances of known miRNA families identified in radish. (**A**) Distribution of known miRNA family member. (**B**) Abundance of each known miRNA family.

To identify known miRNAs in radish leaves at vegetative and reproductive stages, the unique sequences were aligned to the mature sequences of known miRNAs deposited in miRBase. A total of 75 conserved miRNAs representing 21 miRNA families were identified in the both libraries (Table 2; Supplementary Table S3). Among these some miRNA families, miR156/157 and miR165/166 families had 11 and 9 members, respectively; whereas five families, miR171, miR393, miR395, miR397 and miR408 had only one member (Fig. 2A). Moreover, 19 non-conserved miRNAs belonging to 13 miRNA families were also identified in this study, which comprised fewer members as compared to conserved miRNAs (Fig. 2A). For these non-conserved miRNA families, eight miRNA families had only one member, whereas four miRNA families (miR1885, miR2111, miR400 and miR824) and miR394 had two and three members, respectively. In addition, the secondary structures of these known miRNA precursors were predicted as showed in Supplementary Fig. S2. The average minimal folding free energy (MFE) value of these miRNA precursors was −58.6 kcal mol^−1^, and these precursors length ranged from 69 nt to 689 nt, with an average length of 141 nt (Supplementary Table S3).

In this study, the read numbers of 34 identified known miRNA families varied greatly (Table 2). Of these families, the expression levels of several conserved miRNA families, such as miR156/157, miR165/166, miR167 and miR168, were extremely high (Fig. 2B). miR167 (407,770 reads) and miR408 (740,985 reads) were the most abundant in NAU-VS and NAU-RS libraries, accounting for 23.91% and 43.57% of all known miRNA reads, respectively. On the contrary, most of non-conserved miRNA families showed relatively lower read levels as compared to conserved miRNAs. For instance, miR394 and miR400 families had less than 500 reads in both libraries. Moreover, a few non-conserved miRNA families including miR535, miR5227, miR6273 and miR6284, were identified and observed in only one library. Furthermore, different members of each miRNA family displayed drastically different expression levels (Supplementary Table S3).

### Identification of potential novel miRNAs in radish leaf

A total of 44 potential novel miRNAs belonging to 28 novel miRNA families were identified (Table 3; Supplementary Table S4). Among them, one member was detected for 24 novel miRNA families; whereas four families, rsa-miRn2, rsa-miRn7, rsa-miRn15 and rsa-miRn26 were generated from two loci. Secondary structures of these novel miRNA precursors were predicted and identified (Supplementary Fig. S2), with the average MFE value of −46.09 kcal mol^−1^. The average length of these miRNA precursors was 134 nt, with a range of 72 nt to 340 nt. According to the strict criteria of the presence of miRNA* being a prerequisite for predicting novel miRNAs^40^, 12 novel miRNAs were identified with complementary miRNA*s (Supplementary Table S4), which provide strong evidences for these predicted novel miRNAs in radish.

### Identification of bolting related miRNAs

**Figure 3:**
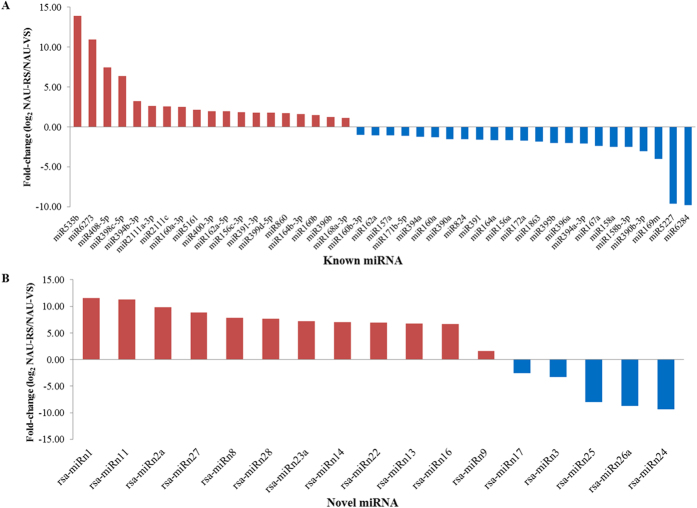
Validation and comparative relative expression of differentially expressed known (A) and novel (B) miRNAs between NAU-VS and NAU-RS libraries.

To identify differentially regulated miRNAs related to regulation of bolting and flowering time in radish, a total of 94 known and 44 novel miRNAs were subjected to differential expression analysis between NAU-VS and NAU-RS libraries. In all, 59 differentially expressed miRNAs which included 42 known and 17 novel miRNAs were identified and considered as bolting related miRNAs (Supplementary Table S5). These miRNAs showed significantly differential expression between the two libraries. Among these miRNAs, 31 miRNAs including 19 known and 12 novel miRNAs were up-regulated in NAU-RS library, whereas 28 miRNAs containing 23 known and five novel miRNAs showed down-regulated patterns (Fig. 3). Furthermore, the expression profiles of different members in the same miRNA family were not identical. Two miRNAs, miR535b (13.93-fold) and rsa-miRn1 (11.52-fold), exhibited the greatest change in expression levels. For differentially expressed novel miRNAs, 11 of 17 were detected in only one library (Fig. 3B; Supplementary Table S5), indicating that these miRNAs might display stage-specific expressions in particular developmental process of radish. These results suggest that differentially regulated miRNAs might be important regulators in the process of radish bolting and flowering.

### Target prediction of bolting and flowering related miRNAs

**Figure 4:**
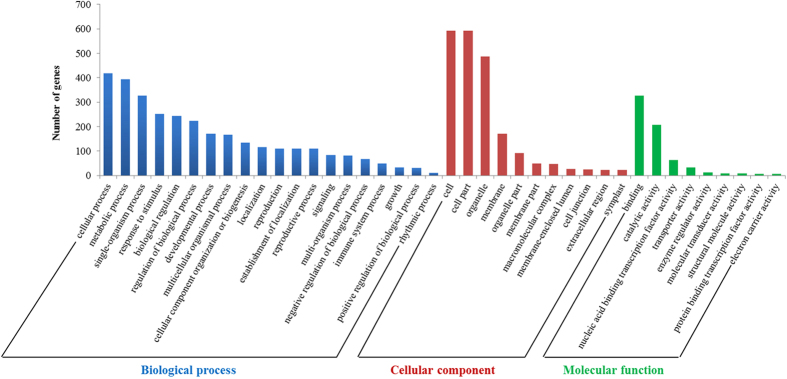
GO classification of target genes for all identified miRNAs in radish.

Target validation is a prerequisite to characterize functionally the biological roles of miRNAs in plants. The psRNATarget server has been proven to be a useful approach to systematically identify target transcripts for known and novel miRNAs^41^. In this study, a total of 3,335 target transcripts with 3,829 target locations were predicted for all the identified miRNAs in radish by the psRNATarget server^41^ (Supplementary Table S6). To better understand the biological functions of miRNAs, the annotation of these target transcripts was performed by Blast2GO analysis. GO analysis revealed that these target genes could be classified into 20 biological processes, 11 cellular components and nine molecular functions (Fig. 4). For biological processes, cellular, metabolic and single-organism processes were the three most dominant GO categories. With regard to cellular components, the three most abundant GO terms were cell, cell part and organelle. The three most dominant GO terms in molecular functions were binding, catalytic activity and nucleic acid binding transcription factor activity. Additionally, GO enrichment analysis demonstrated that these target transcripts were involved in various metabolic and reproductive processes (Supplementary Fig. S3).

In total, 154 target transcripts related to bolting and flowering were identified as the putative targets of 50 bolting-related miRNAs including 27 conserved, 11 non-conserved and 12 novel miRNAs (Supplementary Table S7). Blast2GO analysis of flowering-related miRNAs revealed that these targets could be enriched in 16 biological process, 10 cellular component and nine molecular function categories (Supplementary Fig. S4). Compared with the enriched GO terms of all targets in Fig. 4, the GO analysis results of flowering related targets showed that the great majority of enriched GO categories were consistent with those of all putative targets. The three most abundant GO categories of biological processes and molecular functions were similar to those of all miRNA targets; whereas the ‘cell part’ term which was second enriched in cellular components for all targets, was not found in flowering related targets. In addition, 137 targets were also predicted for unclassified non-conserved miRNAs (Supplementary Table S8).

Many target genes for known miRNAs encoded transcription factors (TFs), such as squamosa promoter binding protein (SBP), NAC domain transcription factor, auxin response factor (ARF) and MYB transcription factor (Table 4). Moreover, some target genes including *SPLs* (targeted by miR156/157)^2^,^3^, *TOC1* (targeted by miR156)^42^, *AP2* (targeted by miR172)^31^,^33^, *SPY* (targeted by miR167)^43^, *AGL16* (targeted by miR824)^44^ and *VRN1* (targeted by miR5227)^45^, were identified to be related to bolting and flowering processes in radish. A few target sequences were also found to be implicated in biotic and abiotic stress response (Supplementary Table S7), such as aluminum-activated malate transporter 9 (*ALMT9*) (Unigene466, miR160) and TIR-NBS-LRR class disease resistance protein (CL9922.Contig3 and Unigene1615, miR1885), which implied that these potential target genes were involved in a broad range of physiological functions and biological processes.

### RT-qPCR validation of miRNAs and their target genes

**Figure 5:**
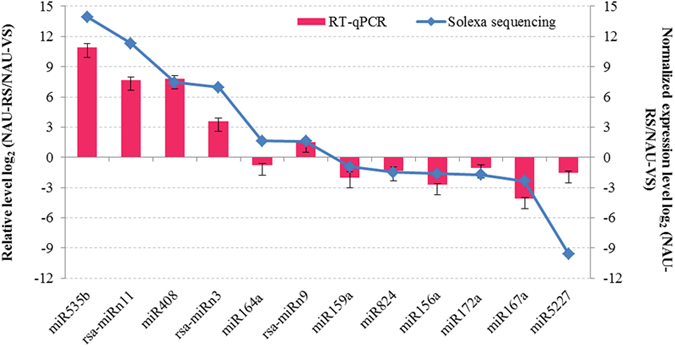
Comparison of relative expression levels of miRNAs between RT-qPCR and Solexa sequencing in radish. Each bar shows the mean ± SE of triplicate assays.

To validate the quality of Solexa sequencing and study the expression patterns of miRNAs in radish, the expression levels of 12 selected miRNAs, miR156a, miR159a, miR164a, miR167a, miR172a, miR408-5p, miR535b, miR824, miR5227, rsa-miRn3, rsa-miRn9 and rsa-miRn11, at vegetative and reproductive phases were validated by RT-qPCR analysis, and were further compared with the results from Solexa sequencing (Fig. 5). The comparisons showed that the expression patterns of most miRNAs shared a similar tendency between the RT-qPCR and NGS sequencing, indicating that the data from sRNA sequencing are reliable and could represent relative expression levels of miRNAs in radish.

**Figure 6:**
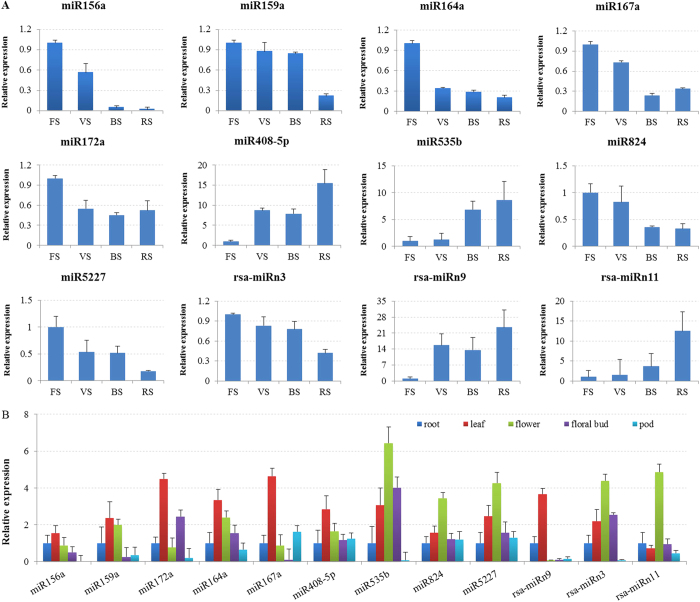
Validation of temporal and spatial expression patterns of miRNAs by RT-qPCR. (**A**) The relative expression levels of miRNAs during different development stages (‘FS’, four-leaf stage; ‘VS’, vegetative stage; ‘BS’, bolting stage; ‘RS’, reproductive stage). (**B**) The relative expression levels of miRNAs in various tissues (root, leaf, flower, floral bud and pod). Each bar shows the mean ± SE of triplicate assays.

**Figure 7:**
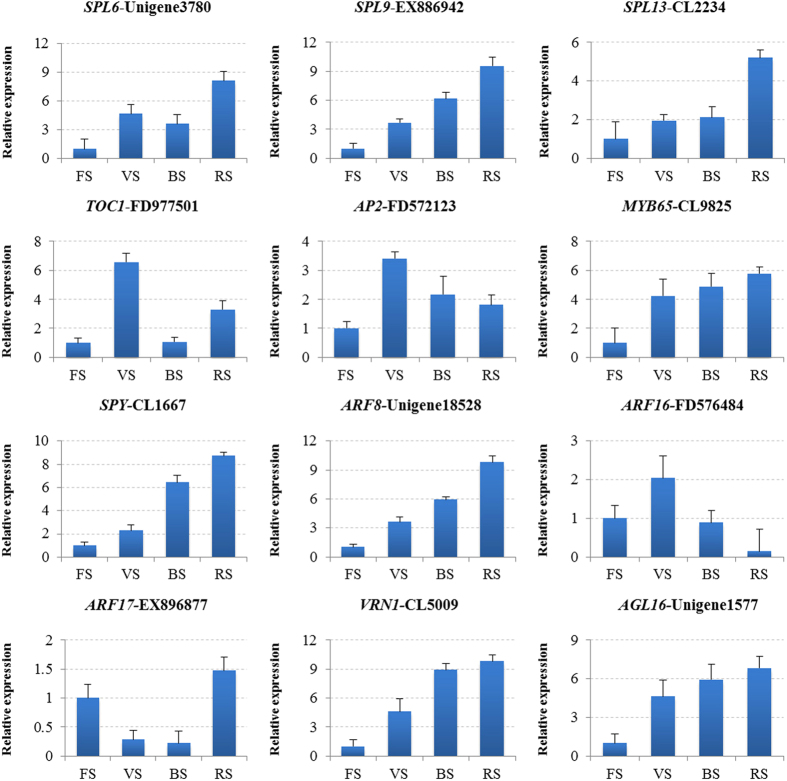
RT-qPCR validation of putative target genes at different development stages. Each bar shows the mean ± SE of triplicate assays.

To investigate the expression patterns of bolting-related miRNAs and their target genes during different developmental stages and in various tissues of radish, 12 miRNAs were selected for RT-qPCR analysis (Fig. 6). As expected, the expression levels of miR156a, miR159a, miR164a, miR167a, miR172a, miR824 and miR5227 peaked at four-leaf stage (FS) and declined from vegetative stage (VS) to reproductive stage (RS) (Fig. 6A); whereas miR408-5p, miR535b, rsa-miRn9 and rsa-miRn11 exhibited similarly higher expression patterns at reproductive stage. As shown in Fig. 6B, miR172a, miR164a and miR167a were most abundant in leaf, whereas miR535b, miR824, miR5227, rsa-miRn3 and rsa-miRn11 had highest expression levels in flower. Moreover, the dynamic expression patterns of 12 corresponding target genes were validated: *SPL6*-Unigene3780, *SPL9*-EX886942, *SPL13*-CL2234 and *TOC1*-FD977501 for miR156a; *AP2*-FD572123 for miR172a; *MYB65*-CL9825 for miR159a; *SPY*-CL1667 and *ARF8*-Unigene18528 for miR167a; *ARF16*-FD576484 and *ARF17*-EX896877 for rsa-miRn11; *AGL16*-Unigene1577 for miR824; *VRN1*-CL5009 for miR5227. The results revealed that the expression of different genes varied at different developmental stages of radish (Fig. 7). For example, four target genes, *SPL13*, *SPL9*, *SPL6* and *TOC1*, being targeted by miR156a showed up-regulated patterns at vegetative stage (Fig. 7). The expression patterns of *SPL13, SPL9* and *SPL6* from vegetative stage to reproductive stage showed negative correlation to the down-regulated miR156a, whereas *TOC1* declined at bolting stage (BS) and remained at relatively higher levels at reproductive stage. These results implied that the different target genes of bolting-related miRNA might play different roles during the transition from vegetative stage to flowering. However, most of miRNAs could negatively regulate their corresponding target genes, which might play important roles during the bolting and flowering processes of radish.

## Discussion

The timely transition from vegetative growth to reproductive development is regulated by a series of flowering-related genes involved in a complex gene-network, which is controlled by diverse environmental and endogenous cues coordinately^1^,^4^,^22^. Previous evidences have suggested that miRNAs were important players in gene regulatory networks involved in plant growth and developmental timing^22^,^46^,^47^. Notably, a number of miRNAs and target genes associated with flowering-time regulation and flower development have been recently identified in some species^17^,^23^,^24^. However, few studies on the miRNA-directed flowering regulatory mechanism in vegetable crops were conducted, and there has been no report on the identification and comparative profiling of bolting- and flowering-related miRNAs and targets in radish.

### Overview of sRNA sequencing in radish leaf

Progress in the next-generation sequencing technology has offered a powerful tool to identify a comprehensive set of miRNAs at certain tissues or during particular stages and to explore the molecular basis of miRNA-mediated flowering-time regulation^8^,^9^. In this study, two sRNA libraries from radish leaves were constructed during vegetative and reproductive stage and sequenced by NGS sequencing. 21-nt sRNA groups were predominant, which was consistent with the reports on tissue-specific miRNAs in *B. oleracea*^48^, wild rice^24^ and castor bean^49^, implying that 21 nt miRNAs may be canonical miRNAs in plants^8^,^24^.

Recently, many studies have shown that most of known miRNAs in the plant kingdom are evolutionarily conserved and highly or moderately expressed^17^,^19^,^20^. In the current study, some conserved miRNA families including miR156/157 and miR168, were sequenced more than 10,000 or even 100,000 times per library, whereas a few non-conserved miRNAs showed relatively lower reads with less than 100, which was in agreement with the previous observations that conserved miRNAs represented higher expression abundance as compared to non-conserved miRNAs^17^,^20^,^23^. In addition, many novel miRNAs were identified in both libraries, and the read numbers of most novel miRNAs were much lower than those of conserved miRNAs, implying that highly conserved miRNAs might play important roles in plant growth and developmental processes^17^.

### Characteristics of bolting-related miRNAs and target genes in radish

High-throughput sequencing in combination with bioinformatics analysis facilitate the identification of differentially expressed miRNAs and functional analysis of miRNAs, which contribute greatly to the elucidation of underlying mechanisms in regulating biological processes and developmental pathways^8^,^50^. Recently, an increasing number of studies reported that many flowering-related miRNAs have been identified in some species, which could facilitate the exploration of miRNA roles in flowering control in plants^23^,^24^. In wild rice, most of identified flowering-related miRNAs, including miR156/157, miR164 and miR167, were found to be down-regulated during the phase change from vegetative stage to flowering stage, whereas miR319 and miR408 showed up-expressed patterns^24^. In trifoliate orange, miR156/157 and miR159 were significantly down-expressed during adult development process in the mutant plant^23^. Several miRNAs related to flower development have also been detected in hickory^17^, *X. sorbifolia*^25^ and andromonoecious poplar^26^, which enriched our knowledge of the regulatory roles of miRNAs. In this study, a set of known and novel miRNAs were identified to be bolting- and flowering-related miRNAs (Supplementary Table S5). The expression patterns of several selected miRNAs which were validated by RT-qPCR analysis, suggested that some previously reported flowering-related miRNAs also showed significantly differential expression in the process of radish bolting and flowering. As expected, the expression level of miR156a was declined from four-leaf stage to reproductive stage, and the level was also relatively low at different tissues during radish flowering phase, which was consistent with several previous studies^2^,^3^,^51^,^52^. In addition, RT-qPCR analysis revealed that several conserved miRNAs including miR156, miR164 and miR167, were down-regulated, which agreed with the results from wild rice^24^; however, their expressions were partly different from the observations in hickory^17^, which maybe result from that hickory belongs to perennial plants which could initiate flowering more than once during their life cycle, whereas it should undergo a long juvenile phase before flowering. Moreover, the majority of selected miRNAs expression patterns exhibited inverse correlation with their corresponding mRNAs, indicating that these miRNAs could negatively regulate their corresponding target genes^19^,^21^,^24^.

Previous studies showed that plant miRNAs could play certain roles in almost all developmental processes by regulating their corresponding target genes^11^, most of which encode key regulatory proteins or TFs that are involved in multiple plant biological processes^8^. In this study, a series of rsa-miRNAs were found to target the genes of TF families, such as *SPLs*, *NACs*, *ARFs*, *WRKYs* and *MYBs*, most of which have been verified to play vital roles in the regulation of plant flowering time. *ARFs* were confirmed to play important roles in plant growth and flower development^53^. Previous studies demonstrated that *ARF8* played essential roles in plant hormone homeostasis^54^ and could promote floral development^16^,^17^. In this investigations, three members of *ARF* gene family, *ARF8*, *ARF16* and *ARF17*, were identified as putative targets of rsa-miR167, rsa-miR160 and rsa-miRn11, respectively, implying that they could participate in the process of radish flowering and flower development. In addition, miR393 have been shown to be correlated with auxin-signaling pathway, which increased level displayed hyposensitivity to auxin and promoted flowering in rice^37^. In this study, rsa-miR393 was predicted to target two genes *TIR1* and *AFB3*, which encoded auxin receptor proteins and could be involved in auxin signal transduction and floral transition. Furthermore, these identified target genes for bolting- and flowering-related miRNAs have shown that some genes could not only influence the floral transition, but also be associated with a wide range of biological functions including signal transduction, defense responses and circadian clock regulation. The result suggested that these bolting- and flowering-related miRNAs and potential target genes were involved in diverse biological and developmental processes in radish.

### MiRNA-mediated flowering-time regulatory networks in radish

**Figure 8:**
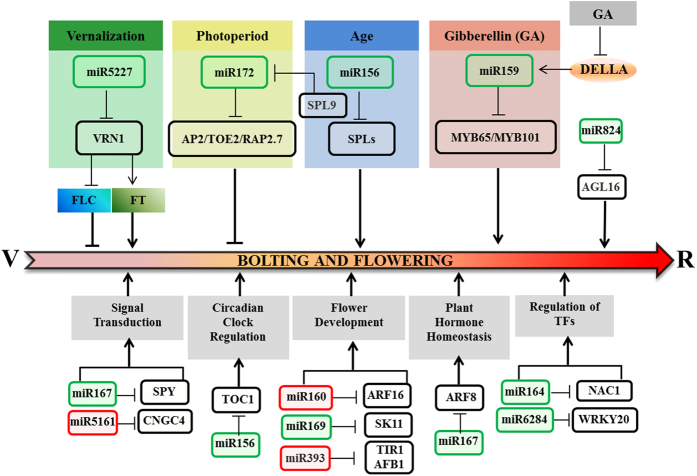
The putative schematic model of miRNA-mediated bolting and flowering regulatory network in radish. ‘V’ and ‘R’: The vegetative and reproductive stage of radish, respectively. The up- and down-regulated miRNAs are in red and green, respectively. All the identified targets are represented in black.

As major regulators of gene expressions, miRNAs regulate the expressions of specific target genes mainly at the post-transcriptional level^6^,^7^. Recently, several flowering related miRNAs and their target genes have been shown to play important roles in the developmental switch from vegetative growth to flowering^4^,^22^,^27^. In this study, several crucial bolting and flowering related genes, such as *SPLs*, *AP2s* and *MYBs*, were identified and involved in the complex genetic networks of bolting and flowering time control. Based on the identification and characterization of *R.sativus* miRNAs and corresponding target genes, a putative schematic model of miRNA-mediated bolting- and flowering-time regulatory networks was proposed (Fig. 8). Considerable reports showed that miR156 targeted 11 *SPL* genes in *Arabidopsis*, which controlled the newly defined age pathway and acted in regulating flowering time by the crosstalk with photoperiodic, GA and vernalization pathways^2^,^3^. The roles of miR156-SPLs node in controlling flowering time could be pronounced by the evidence that the overexpression of miR156 resulted in delayed flowering in several species^28^,^29^,^30^. Remarkably, the expression of miR156 was temporally regulated by plant age^2^,^51^, which was higher in embryos and seedlings and displayed a lower level at reproductive phase^52^. In agreement with previous studies, both NGS sequencing and RT-qPCR validation revealed that rsa-miR156 was down-expressed at reproductive stage of radish, which was found to target a series of *SPL* genes including *SPL3*, *SPL5*, *SPL6*, *SPL9*, *SPL13* and *SPL15* (Table 4), inferring that rsa-miR156 and its *SPL* targets could play important roles in determining bolting and flowering time in radish (Fig. 8).

Interestingly, a vital floral integrator miR172, which is important in regulating the developmental transition of flowering and the determination of floral organ identity, was down-regulated by miR156 via *SPL9*^27^,^46^,^51^. The putative target gene of miR172 was a small group of *AP2*-like genes, including *AP2*, *TARGET OF EAT1* (*TOE1*), *TOE2*, *TOE3*, *SCHLAFMUTZE* (*SMZ*) and *SCHNARCHZAPFEN* (*SNZ*), which mainly function as flowering repressors^31^,^32^,^33^. In this study, rsa-miR172 was identified and predicted to target three flowering genes, *AP2*, *TOE2* and *RAP2.7*, which would be down-regulated by rsa-miR172 and regulate flowering time (Fig. 8).

Functions in flowering time control have been shared by miR159 and its target MYB transcription factors via the GA pathway. It had been shown that GA treatment could direct the degradation of DELLA proteins that cause an increase in miR159 level^22^,^47^. MiR159-MYB module played vital roles in flowering time manipulation, which had been demonstrated in *Arabidopsis*^34^ and *Sinningia speciosa*^35^. Our results also showed that rsa-miR159 was predicted to target two MYB genes, *MYB65* and *MYB101*. Moreover, two flowering related genes, *AGL16* and *VRN1*, belonging to MADS-box transcription factors were identified for rsa-miR824 and rsa-miR5227, respectively. MiR824 targeted *AGL16* gene could modulate the flowering time of *Arabidopsis* by interacting with *FLOWERING LOCUS C* (*FLC*), *SHORT VEGETATIVE PHASE* (*SVP*) and *FLOWERING LOCUS T* (*FT*)^44^; while *VRN1* as flowering activator repressed the expression of floral repressor *FLC*^45^ and modulated the level of *FT* in a rhythmic manner^55^. These evidences indicated that rsa-miR824 and rsa-miR5227 might be essential components of gene regulatory network that orchestrate developmental transition of flowering in radish (Fig. 8). These findings showed that the expression of bolting- and flowering-related genes including *SPLs*, *AP2s*, *MYBs*, *AGL16* and *VRN1* mediated by corresponding rsa-miRNAs played vital roles in inhibiting or promoting the initiation of radish bolting and flowering, indicating that these identified crucial miRNA-target modules could serve to dissect the genetic networks of miRNA-mediated bolting- and flowering-time control in radish.

In conclusion, bolting-related miRNAs and their targets between vegetative and reproductive stages were first identified and comparatively profiled at transcriptome-wide level using small RNA sequencing in radish. A total of 28 conserved miRNAs, 14 non-conserved miRNAs and 17 novel miRNAs were significantly differentially expressed and identified as bolting- and flowering-related miRNAs in radish. RT-qPCR analysis suggested that some differentially regulated miRNAs displayed differential temporal and spatial expressions. GO categorization and functional analysis suggested that several bolting- and flowering-related miRNAs, such as miR156, miR172, miR159, miR824 and miR5227, targeted some key transcription factors and regulatory proteins which were involved in radish bolting and flowering control. The outcomes of this study provided insights into the miRNA-mediated flowering-time regulatory networks in radish, and advanced the understanding of molecular mechanisms associated with the control of bolting and flowering time in Brassicaceae crops.

## Methods

### Plant Materials

Seeds of an advanced inbred line of radish, ‘NAU-LU127’ (late bolting and flowering), were sown in plastic pots and cultured in greenhouse at 16 h light (25 °C)/8 h dark (16 °C). To examine the temporal and spatial expression patterns of miRNAs, radish leaves were collected during four-leaf stage (FS), vegetative stage (VS), bolting stage (BS) and reproductive stage (RS), respectively. The root, leaf, flower, floral bud and pod were harvested separately at reproductive stage. All the samples were collected from three randomly selected individual plants and immediately frozen in liquid nitrogen and stored at −80 °C for further use.

### High-throughput sequencing of transcriptome and small RNAs

The equal amounts of radish leaves from three independent biological replicates at vegetative and reproductive stages were pooled and used for a radish leaf transcriptome library construction. Total RNA was isolated using Trizol reagent (Invitrogen) according to the manufacturer’s protocol. The transcriptome library was prepared from the mixed leaves using an Illumina TruSeq RNA Sample PrepKit following the manufacturer’s instructions. Two small RNA (sRNA) libraries from leaves at vegetative stage (NAU-VS) and reproductive stage (NAU-RS) were constructed following previously reported procedures, respectively^19^,^20^. Briefly, the sRNAs sized at 18–30 nt were separated and gel-purified on a 15% polyacrylamide denaturing gel from the total RNAs of the two samples. Then the isolated sRNAs were ligated to 5′- and 3′-RNA adaptors by T4 RNA ligase (TaKaRa) and transcribed to single-stranded cDNA using One Step PrimerScript miRNA cDNA Synthesis Kit (TaKaRa). Both small RNAs and transcriptome were sequenced using Illumina HiSeq™ 2000 at Beijing Genomics Institute (BGI), Shenzhen, China.

### Bioinformatic analysis of small RNA sequencing data

After removing the low quality and contaminated reads from raw reads, clean reads were generated from two independent sRNA libraries. The unique small RNAs that ranged from 18 to 30 nt in length were mapped to the radish reference sequences, which were composed of our radish leaf transcriptome library, the available GSS and EST sequences deposited in NCBI databases. These unique sRNA sequences were compared with non-coding sRNAs deposited in the NCBI GenBank and Rfam (RNA family) databases by BLASTn search. Thereafter, these matched non-coding sRNAs including ribosomal RNAs (rRNAs), transfer RNAs (tRNAs), small nucleolar RNAs (snoRNAs), and small nuclear RNAs (snRNAs) were filtered and removed. The remaining matched unique sRNA sequences were then aligned with known miRNA sequences from other plant species deposited in miRBase 21 (http://www.mirbase.org/index.shtml) with a maximum of two mismatches for known miRNA identification. The secondary structures of all known miRNA precursors were obtained by Mfold software^56^.

### Identification of novel miRNAs

After mapping the clean reads to NCBI, Rfam and miRBase databases, the unannotated sRNAs were used for novel miRNAs identification. These sRNA sequences were subjected to MIREAP software (https://sourceforge.net/projects/mireap/) to predict novel miRNA candidates according to the parameters as follows: the allowed miRNA sequence length ranged from 18–25 bp, while the range of reference sequence length was from 20–23 bp. The previously reported basic criteria including the presence of corresponding miRNA* sequences, minimum 16 bp in the miRNA and miRNA* pairs, maximum −18 kcal mol^–1^ for a miRNA precursor free energy, no more than four bulge and asymmetry between miRNA and miRNA*, were also followed in novel miRNA prediction^40^. In addition, according to the instructions for high confidence miRNAs in miRBase 21, the predicted novel miRNAs were further screened and validated in this study. The secondary structures of pre-miRNA for all novel miRNA candidates were constructed by Mfold software^56^.

### Differential expression analysis of bolting related miRNAs

The expression abundance of all the identified miRNAs was normalized to one million against total clean reads of miRNAs in each library (normalized expression = actual miRNA count/total count of clean reads × 1,000,000). The differential expression of bolting-related miRNAs between the two libraries was calculated as: fold-change = log_2_ (miRNA normalized reads in ‘NAU-RS’/miRNA normalized reads in ‘NAU-VS’). The *P*-value was calculated according to previously established methods^17^,^24^,^57^. The miRNAs with fold-change ≥ 1.0 or ≤ −1.0 and *P* ≤ 0.05 were determined as up- or down-expressed miRNAs during radish bolting and flowering, respectively.

### Prediction and annotation of target genes for miRNAs

The potential target prediction of all the identified miRNAs was conducted using the plant small RNA target server (psRNATarget; http://plantgrn.noble.org/psRNATarget/)^41^. The publicly radish EST and GSS sequences in NCBI database and our mRNA transcriptome sequences were used as the radish reference sequences. To investigate the potential functions of these predicted target transcripts, Blast2GO program was performed for Gene Ontology (GO) annotation and enrichment analysis. Candidate targets were analyzed by BLASTX search against the NCBI Nr database with the default parameters. In addition, KEGG Orthology Based Annotation System (KOBAS 2.0; http://kobas.cbi.pku.edu.cn/home.do/) was employed to further understand the biological functions of genes^19^,^58^.

### RT-qPCR validation

Reverse transcription quantitative real-time PCR (RT-qPCR) was performed to validate the quality of high-throughput sequencing and the relative expression levels of miRNAs and targets according to previous methods^20^,^59^. Total RNAs from all samples were isolated and transcribed to cDNA as described above following the manufacturer’s protocol. Each reaction was performed in a total volume of 20 μl containing 10 μl 2 × SYBR green reaction mix, 2.0 μl diluted cDNA and 0.2 μM of each primer. The amplification reactions were carried out on a BioRad iQ5 sequence detection system (BioRad, USA) following the reported protocol^20^. Based on the mature miRNA sequences, the specific forward miRNA primers were designed, and the reverse primers were universal for miRNA. The specific primers of target genes for RT-qPCR were designed using Beacon Designer 7.0 (Premier Bio-soft International, USA). All the primer sequences of PCRs are listed in Supplementary Table S1. For each condition, three replicates were performed and expression levels were normalized according to that of the internal control. The relative gene expression data were analyzed using the 2^−∆∆*C*T^ method^60^. The 5.8S rRNA was used as the reference gene^59^.

## Additional Information

**How to cite this article**: Nie, S. *et al.* Identification of bolting-related microRNAs and their targets reveals complex miRNA-mediated flowering-time regulatory networks in radish (*Raphanus sativus* L.). *Sci. Rep.* **5**, 14034; doi: 10.1038/srep14034 (2015).

## Supplementary Material

Supplementary Information

## Figures and Tables

**Table 1:** Statistical analysis of sequencing reads from NAU-VS and NAU-RS libraries of radish leaves.

Category	NAU-VS		NAU-RS	
	Count	Percentage (%)	Count	Percentage (%)
Raw reads	19,168,474		18,052,596	
High quality	19,048,317	100%	17,949,618	100%
Clean reads	18,948,210	99.47%	17,893,663	99.69%
3′adapter_null	11,289	0.06%	7,163	0.04%
Insert null	7,544	0.04%	1,605	0.01%
5′adapter contaminants	53,199	0.28%	21,828	0.12%
Smaller than 18nt	25,663	0.13%	22,629	0.13%
Poly A	2,412	0.01%	2,730	0.02%

**Table 2:** Known miRNA families and their abundance identified from NAU-VS and NAU-RS libraries.

Family	Members	Counts		Total reads	Ratio (NAU-RS/NAU-VS)
		NAU-VS	NAU-RS		
Conserved					
miR156/157	11	266,846	150,543	417,389	0.56
miR158	3	276,036	47,200	323,236	0.17
miR159	4	1,998	1,746	3,744	0.87
miR160	5	15,531	25,963	41,494	1.67
miR162	4	1,968	2,663	4,631	1.35
miR164	2	25,738	8,908	34,646	0.35
miR165/166	9	291,809	283,516	575,325	0.97
miR167	5	407,770	82,164	489,934	0.20
miR168	4	182,405	162,566	344,971	0.89
miR169	3	1,829	214	2,043	0.12
miR171	1	2,940	1,276	4,216	0.43
miR172	5	80,912	26,261	107,173	0.32
miR390	3	6,814	2,257	9,071	0.33
miR391	2	25,775	9,225	35,000	0.36
miR393	1	22	13	35	0.59
miR395	1	681	160	841	0.23
miR396	3	5,086	6,554	11,640	1.29
miR397	1	854	633	1,487	0.74
miR398	2	411	30,067	30,478	73.16
miR399	5	592	1,129	1,721	1.91
miR408	1	4,544	740,985	745,529	163.07
Non-conserved					
miR1885	2	8,337	12,369	20,706	1.48
miR1863	1	9,168	2,416	11,584	0.26
miR2111	2	113	638	751	5.65
miR394	3	72	55	127	0.76
miR400	2	130	241	371	1.85
miR403	1	1,900	1,485	3,385	0.78
miR535	1	0	2,797	2,797	–
miR5161	1	1,423	5,868	7,291	4.12
miR5227	1	144	0	144	0
miR6273	1	0	360	360	–
miR6284	1	163	0	163	0
miR824	2	1,822	706	2,528	0.39
miR860	1	212	653	865	3.08

**Table 3:** Novel miRNAs and their abundance identified from NAU-VS and NAU-RS libraries.

miRNA	Mature sequence (5′-3′)	Size	LP	MFE	miRNA reads		Total miRNA reads	Total miRNA* reads	loci
					NAU-VS	NAU-RS			
rsa-miRn1	AGAAGAGGAAGAGGATGAAGAT	22	209	−75.8	0	525	525	–	1
rsa-miRn2	AGGGGAGGATGGGTGGGTTTC	21	106	−44.2	0	168	168	–	2
rsa-miRn3	CATTGACTGTATGCATTGGGAG	21	272	−73.2	919	86	1,005	1	1
rsa-miRn4	TCGGAATTCCGTCGGAATATA	21	100	−56.31	743	1,402	2,145	25	1
rsa-miRn5	TACCGATAGATGTGGAAGCGT	21	184	−75.8	2,502	4,596	7,098	41	1
rsa-miRn6	TTTGCGTGAGTATGTGGATGT	21	119	−49	2,809	1,341	4,150	35	1
rsa-miRn7	AGCAAACGAGAATTGAACGGA	21	106	−47.24	842	1,018	1,860	4	2
rsa-miRn8	ATGGCCTTTATATCGTATTCGAA	23	109	−30.2	0	40	40	8	1
rsa-miRn9	ATGTGGGATGTGATTGTCAAG	21	83	−22.36	112	319	431	–	1
rsa-miRn10	GTGACCGGCGCGTGGCGGCTC	21	74	−36.9	15	9	24	–	1
rsa-miRn11	ATGCCTGGCTCCCTGTATGCC	21	106	−49.3	0	457	457	–	1
rsa-miRn12	GGTAGTTTGACCGCGAAATTT	21	143	−28	6,902	11,143	18,045	–	1
rsa-miRn13	GTCTGTATGGTATGGGTGGAGG	22	110	−52.11	0	19	19	–	1
rsa-miRn14	GCTAATGAGATCGAAATACTGA	21	182	−28.9	0	23	23	1	1
rsa-miRn15	CAGAGACAGAGAGGAGAAAGGAA	23	90	−34	22	28	50	–	2
rsa-miRn16	ATATACTGAAGTTTATACTCT	21	208	−37	0	18	18	–	1
rsa-miRn17	TATGAATGATGCGGGAGATGT	21	112	−33.5	108	17	125	6	1
rsa-miRn18	AGGACCAGGTCGACGACGCCG	21	80	−44.9	312	558	870	98	1
rsa-miRn19	TGAGCGGCAACTATTGTAGGT	21	111	−50.7	14	9	23	–	1
rsa-miRn20	TCTTGAGTTCGAGGGACGCCA	21	107	−65.6	90	153	243	3	1
rsa-miRn21	GTTGGGATCGCTTGTTGGAGT	21	340	−104.4	798	0	798	–	1
rsa-miRn22	AGAAGAACGGGAACAAAGAAA	22	147	−23.6	0	22	22	–	1
rsa-miRn23	ACGAGGCTGTGGCTTACGGTG	21	111	−37.1	0	26	26	1	1
rsa-miRn24	AGGATTGAGTCTAGAAGCATA	21	270	−57.1	127	0	127	–	1
rsa-miRn25	AGAACGATATAAAAGATCATGG	22	107	−30.2	48	0	48	20	1
rsa-miRn26	GAGGGGAGGATGGGTGGGTTTC	22	106	−44.2	82	0	82	–	2
rsa-miRn27	GCGTCCCCGACATGGTCGTCT	21	72	−31.7	0	81	81	5	1
rsa-miRn28	GAAGATTTAGTAGAGTTGGCG	21	113	−27.3	0	37	37	–	1

*LP (nt):* The length of precursor, *MFE (kcal mol^–1^):* minimal folding free energy.

**Table 4:** Identified targets for conserved miRNAs in radish.

miRNA family	Target sequence	Target gene	Target gene annotation
miR156/157	CL289.Contig1	*SPL5*	Squamosa promoter-binding-like protein 5
	CL2234.Contig1	*SPL13*	Squamosa promoter-binding-like protein 13
	Unigene3780	*SPL6*	Squamosa promoter-binding-like protein 6
	Unigene9933	*SPL15*	Squamosa promoter-binding-like protein 15
	Rsa#S43017568	*SPL3*	Squamosa promoter-binding-like protein 3
	FD977501	*TOC1*	Two-component response regulator-like APRR1
	EX886942	*SPL9*	Squamosa promoter-binding-like protein 9
miR158	Rsa#S42049270		Pentatricopeptide repeat-containing protein
miR159	CL3756.Contig1	*KOM*	Protein KOMPEITO
	CL2461.Contig3	*UVH6*	DNA repair helicase UVH6
	CL8717.Contig1	*SPL*	Putative transcription factor SPL
	CL9825.Contig1	*MYB65*	MYB domain protein 65
	Rsa#S42037487	*MYB101*	MYB domain protein 101
	Rsa#S42034459	*UBC17*	Putative ubiquitin-conjugating enzyme E2 17
miR160	Unigene466	*ALMT9*	Aluminum-activated malate transporter 9
	Rsa#S42581764	*ARF16*	Auxin response factor 16
	EW732793	*PMEI1*	Pectin methylesterase inhibitor AtPMEI1
miR164	Unigene2996	*NAC1*	Transcription factor NAC1
	Rsa#S43010415	*HRE1*	Ethylene-responsive transcription factor ERF073
	EW715661	*NAC100*	NAC domain containing protein 100
miR165/166	CL7974.Contig1	*ATHB-15*	Homeobox-leucine zipper protein ATHB-15
miR167	CL1667.Contig1	*SPY*	putative UDP-N-acetylglucosamine-peptide N-acetylglucosaminyltransferase SPINDLY
	Unigene18528	*ARF8*	Auxin response factor 8
miR169	CL1568.Contig1	*SK 11*	Shaggy-related protein kinase alpha
	FY429716	*SIP2*	Putative galactinol-surose galactosyltransferase
	EX895539		Hypothetical protein
miR171	CL5811.Contig1	*HAM3*	Protein LOST MERISTEMS 3
miR172	CL2600.Contig1	*RAP2.7*	TARGET OF EARLY ACTIVATION TAGGED 1
	CL8288.Contig2	*TPS8*	Putative alpha,alpha-trehalose-phosphate synthase [UDP-forming] 8
	Rsa#S41997500	*SR33*	SC35-like splicing factor 33
	EW732550	*TOE2*	AP2-like ethylene-responsive transcription factor TOE2
	FD572123	*AP2*	Floral homeotic protein APETALA 2
	EW719579	*SCL30A*	SC35-like splicing factor 30A
miR391	CL175.Contig3	*ACA10*	Calcium-transporting ATPase 10
miR393	Unigene359	*AFB3*	Auxin signaling F-box 3 protein
	FD955493	*TIR1*	Protein TRANSPORT INHIBITOR RESPONSE 1
	Rsa#S43016969	*GRH1*	GRR1-like protein 1
miR395	FY441630	*APS4*	Sulfate adenylyltransferase
miR396	CL6202.Contig1	*ACHT5*	Atypical CYS HIS rich thioredoxin 5
	Unigene4815	*SMC5*	Structural maintenance of chromosomes 5
miR397	CL379.Contig2	*scpl48*	Serine carboxypeptidase-like 48
miR399	Unigene10472	*CNGC11*	Cyclic nucleotide-gated channel 11
miR408	CL13722.Contig2	*DMP7*	DUF79 domain membrane protein 7
	CL13722.Contig5		DNAJ heat shock family protein
