# Supplementary material for: Identification of bolting-related microRNAs and their targets reveals complex miRNA-mediated flowering-time regulatory networks in radish (Raphanus sativus L.)
Source: Sci Rep. 2015 Sep 15;5:14034. doi: 10.1038/srep14034 (PMC4570191; doi:10.1038/srep14034)
Supplement: Supplementary Information [file srep14034-s1.pdf]

Supplementary information

Identification of bolting-related microRNAs and their targets reveals complex miRNA-mediated flowering-time regulatory networks in radish (*Raphanus sativus* L.)

Shanshan Nie, Liang Xu, Yan Wang, Danqiong Huang, Everlyne M. Muleke, Xiaochuan Sun, Ronghua Wang, Yang Xie, Yiqin Gong & Liwang Liu*

1. Supplementary Figures

Supplementary Figure S1: Venn chart for total sRNAs (A) and unique sRNAs (B) between NAU-VS and NAU-RS libraries.

Supplementary Figure S2: The secondary structures of *R. sativus* known and novel miRNA precursors. The mature miRNAs are in red and miRNA*s in blue. (“.” represent base mismatches, “(” represent base matches).

Supplementary Figure S3: GO enrichment multiple analysis in the classification of biological process.

Supplementary Figure S4: GO classification of target genes for bolting and flowering-related miRNAs in radish.

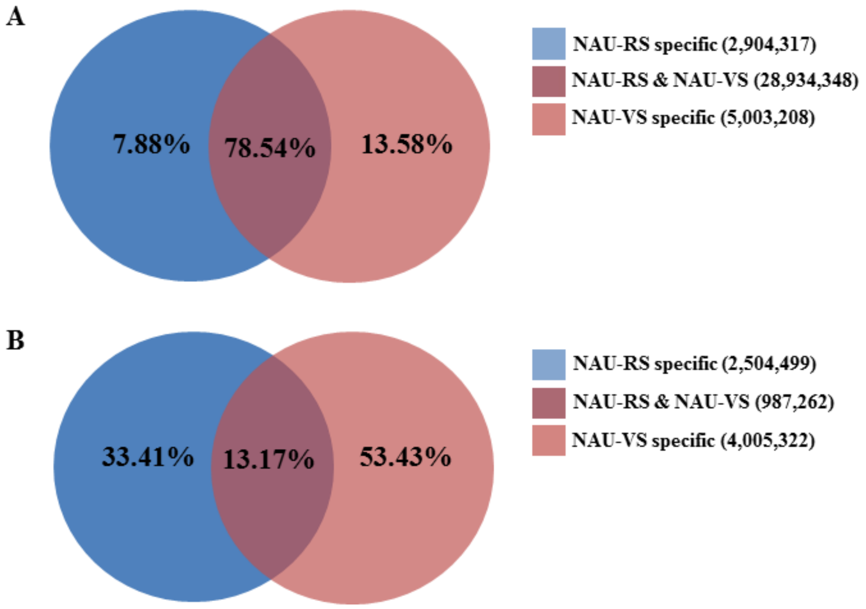

Supplementary Figure S1: Venn chart for total sRNAs (A) and unique sRNAs (B) between NAU-VS and NAU-RS libraries.

Supplementary Figure S2: The secondary structures of *R. sativus* known and novel miRNA precursors. The mature miRNAs are in red and miRNA*s in blue. (“.” represent base mismatches, “(” represent base matches).

Conserved miRNAs

miR156

GAAATGCAACAGAGAAAAACTGACAGAAGAGAGTGAGCACATGCAGGCACTGTTATGTCTCTATAACTTTGCGTGTGCGTGCTCACGGCTCTTTCTGTCAGTTGCCTGACTTCTCGCTTTCT

((((.((....(((((.((((((((((((((.((((((((..(((.(((..((((((....))))))..))).)))..))))))))..))).))))))))))).......))))))))))).

*******************CTGACAGAAGAGAGTGAGCAC**

TGCTCACGGCTCTTTCTGTCAGT*****************

miR156a

ACATAGCAACTGACAGAAGAGAGTGAGCACACAAAGGTAATTTGCATACTGCATTTGCTTCTCTTGCGTGCTCACTGCTCTCTCTGTCAGATTCTAGTGCTGATC

...(((((.(((((((((((((((((((((.(((((((((..(((.....))).))))))...))).))))))))).)))).)))))))).......)))))...

**********TGACAGAAGAGAGTGAGCAC***

miR156c

TGAGAAAATGTTGACAGAAGAAAGAGAGCACAAGCCGGGATTAGCAAAAAGATTAGTTTTGCCTTTGTCGGGAGTGTGCTCTCTTTACTTCTGCCACCATCGCACAC

.......(((.((.((((((((((((((((((..((((.(...((((((.......))))))...).))))...)))))))))))).)))))).)).)))).......

**********TTGACAGAAGAAAGAGAGCAC**

miR156c-3p

GCATAGAAACTGACAGAAGAGAGTGAGCACACAAAGGCACTTTGCATGTTCGATGCATTTGCTTCTCTTGCGTGCTCACTGCTCTATCTGTCAGATTTCGGC

((...(((((((((((((((((((((((((.((((((((...(((((.....)))))..)))))...))).))))))))).)))).)))))))).)))).))

GCTCACTGCTCTATCTGTCAGA****

miR156d

GGATCTATTGTTGACAGAAGAGAGGGAGCACCAGCAGCCACCAGCTCTAGAGAGGCTTTTGGGTGCTTGTGCTCTCTATTCTTCTGCCATCAATCCGACTCGG

(..((.((((.((.(((((((.(((((((((.((((.(((..(((((....)).)))..))).)))).)))))))))..))))))).)).))))..))..)..

**********TTGACAGAAGAGAGGGAGCAC**

miR156g

GATAGTGTTGACAGAAGATAGAGAGCACAGATGATGCGATGCAATTTGGAGCAGCTTTTATGCATCATACTCCTTTGTGCTCTCTAGTCTTCTGTCATCACTCGT

...((((.(((((((((((((((((((((((.((((.((((((....((.....))....)))))).)).)).)))))))))))).))))))))))).))))...

**********ACAGAAGATAGAGAGCACAGA**

miR156k

GAAAAAGAAGTTGACAGAAGAGAGTGAGCACACAAAGGGGGAATTGTATAAAATTAAGTTTGTTGTATATGGTTGCTTTTGCGTGCTCACTCTCTTTATGTCACAACTTCTCCTTCA

(((..((((((((((((((((((((((((((.((((((.((...(((((((((......)).)))))))...)).)))))).))))))))))))))).)))))..))))))..))).

**********TTGACAGAAGAGAGTGAGCAC**

miR156k-5p

GAAAAGAAAGTGACAGAAGAGAGCGAGCACACAAAGGGAGAATTGTATAAAATTAATTTGTATATGGTCACTTTTGCGTGCTCATTCTCGTTCTGTCATAACTTTCTCCTTCA

(((.(((((((((((((((((((.((((((.((((((((....((((((((.....))))))))...)).)))))).)))))).))))).)))))))...)))))))..))).

**********TGACAGAAGAGAGCGAGCAC***

miR157a

GTGTTGACAGAAGATAGAGAGCACAGATGATGAGATACAATTCGGAGCATGTTCTTTGCATCTTACTCCTTTGTGCTCTCTAGCCTTCTGTCATCACC

(((.(((((((((.(((((((((((((.((((((((.(((...((((....)))))))).)))))).)).)))))))))))))..)))))))).))).

***TTGACAGAAGATAGAGAGCAC**

miR157a-3p

GAGGCATTGAGAGTGTTGACAGAAGATAGAGAGCACAGATGATAAGATACAATTCGGAGCAGTTTCTTTGCATCTTACTCCTTTGTGCTCTCTAGCCTTCTGTCATCACCTTTTATTTGCTTT

((((((.((((((((.(((((((((.(((((((((((((.((((((((.(((...((((....))))))).)))))).)).)))))))))))))..))))))))).)).))))))..))))))

GCTCTCTAGCCTTCTGTCATC**************

miR157d

TGCTGGTTGTTGACAGAAGATAGAGAGCACTGAGGATTACATGCAAGTACATATATATGTATCATCATCACACCCGCCTGTGGATGATAAAAGATGTGAAACAAATTCAAGAGAGAGAGAGAACGAGAATGA
GAAAGAGCCTGCATGTCTACTCTTTTGTGCTCTCTATACTTCTGTCACCACCATTA

...((((.((.(((((((((((((((((((.(((((..((((((.((.(((((.....(((((((..((((......))))))))))....)))))).......(((......))).................
......)).)))))))....))))).))))))))))).)))))))))).))))...

**********TGACAGAAGATAGAGAGCAC**

****

miR158a

TTCTCGGTATTTCTTTGTTTATATTTTTGGAAAAAGCGATGAGGTTTCGTTGCACTTTCCCAAATGTAGACAAAGCAAAACCGTGAT

.((.((((.((.((((((((((((((..(((((..(((((((....)))))))..))))).)))))))))))))).)).)))).)).

TCCCAAATGTAGACAAAGCA*******

miR158b

ATCTCTGTGCTTCTTTGTCTACACTTTTGGAAAAGGTGATGATATCATTGCTTTTCCCCAAATGTAGACAAAGCAATACCGTGAT

(((.(.(((...(((((((((((..(((((.(((((..(((....)))..)))))..))))))))))))))))...))).).)))

CCCCAAATGTAGACAAAGCA*******

miR159a

GTAGAGCTCCTTAAAGTTCAAACATGAGTTGAGCAGGGTAAAGAAAAGCTGCTAAGCTATGGATCCCATAAGCCCTAATCCTTGTAAAGTAAAAAAGGATTTGGTTATATGGATTGCATATCTCAGGAGCTT

TAACTTGCCCTTTAATGGCTTTTACTCTTCTTTGGATTGAAGGGAGCTCTAC

(((((((((((..((((((((...((((.(((((((((((...((((((.((.((.((((.(..(((((((((..(((((((..........))))))).)))).)))))..).)))).)).)).)))))

)...)))))))......))))..))))...))))))))..)))))))))))

****

*****************************TTTGGATTGAAGGGAGCTCTA*

miR159a-5p

AACAAAAAAAGATTGAAGTAGAGCTCCTTTAAGTTCAAACGAGAGTTTAGTAGGGTAAAGAAAATCTGCTAAGCTATGGATCCCATAAGCCCTAATCCTTATAGAGATTGAAAAGGATTTGGTTATATGGCT

TGCATATCTTAGGAGCTTTAACTTGCCCTTTAATTGCTTTTACTCTTCTTTGGATTGAAGGGAGCTCTACATCTTTTTTTCTATTCA

...((((((((((....((((((((((((.((((((((.((((((..((((((((((...(((.((.(((((.((((.(..(((((((((..(((((((............))))))).)))).)))))..

).)))).))))).)).)))...)))))))......)))...)))))).)))))))).)))))))))))))))))))))).......

*********************GAGCTCCTTTAAGTTCAAACG**

****

miR159b

CTGATTAGGGTTAAGAAGTGGAGCTCCTTGAAGTCCAATAGAGGTTCTTGCGTGGGTAGATTGAGCTGCTGAGCTATGGATCCCTCAGCCCTATCCCATCATAAAGAGTAGTCCTCTTGGATAGGCTTGTGG

CTTGCATATCTCAGGAGCTTTATTACCACATATGTTAGATCCTTGTTTGGATTGAAGGGAGCTCTACATCTAAATCTTTCAC

.(((..((((((.(((.((((((((((((..(((((((..((((.(((.(((((((((((...(((((.(((((.((((.(..((.(((.(((((((.......(((((.....)))))))))))).))).))

..).)))).))))).)))))..)))))...))))).))).))))..)))))))..))))))))))))).))).))))))))).

****

CTTGCATATCTCAGGAGCTT**

miR159c-5p

TAGCCAAAGGAGCTCCCTTCGTCCAAAACGAGGAGGACACGTTTTAAGGAAATAAGAAAACCGAAAATCTAAGTGTTCATGGATTCCCCGTATGAAGTGTGTTAGGTTAATACACCTTGAAGAATCCGCACT

TTAAGGGGGTTTGCATGGGCTCTTAGATTTTCTCTCTTTCTCTCTTTATATGTATATCCTTTTCTTCTCTTCGTTTGGATTGAAGGGAGCTCCTTTTTTT

.....(((((((((((((((((((((.(((((((((.((((..((((((....(((((...(((((((((((.(((((((.(..((((.(.(((((((((..((((......)))).........))))))

)))).))))..).))))))).))))))))))))....))))).)))))).))))..........))))))))))))))).)))))))))))))))).....

***********AGCTCCCTTCGTCCAAAACG***

****

miR160a

TGATGTATTATATATGTATATGCCTGGCTCCCTGTATGCCATACACTAAGTTCATCGACCATCGGCGACCTCCGTGGATGGCGTATGAGGAGCCAAGCATATTCTCAGCATAGACATATTT

..((((.....(((((((((((.(((((((..(((((((((.(((..((.((..((.....))..)).))..))).)))))))))..))))))).))))))).....))))).))))....

********************TGCCTGGCTCCCTGTATGCCA**

GCGTATGAGGAGCCAAGCATA******************

miR160a-3p

TGATGTATTATATATGTATATGCCTGGCTCCCTGTATGCCATACACTAAGTTCATCGACCATCGGCGACCTCGGTGGATGGCGTATGAGGAGCCAAGCATATTCTCAGCATAGACATATTT

..((((.....(((((((((((.(((((((..(((((((((.(((..((.((..((.....))..)).))..))).)))))))))..))))))).))))))).....))))).))))....

********************TGCCTGGCTCCCTGTATGCCA**

GCGTATGAGGAGCCAAGCATA******************

miR160b

TGATGTATTATATATGTATATGCCTGGCTCCCTGTATGCCATACACTAAGTTCATCGACCATCGGCGACCTCCGTGGATGGCGTATGAGGAGCCAAGCATATTCTCAGCATAGACATATTT

..((((.....(((((((((((.(((((((..(((((((((.(((..((.((..((.....))..)).))..))).)))))))))..))))))).))))))).....))))).))))....

*******************ATGCCTGGCTCCCTGTATGCC***

GCGTATGAGGAGCCAAGCATA******************

miR160b-3p

CATATATACATAGTACATATATGCCTGGCTCCCTGTATGCCATACACTAAGCTCATCGACCATCGAGGACTTACGTGGATGGCGTACAGAGTAGTCAAGCATGTTTTCATATGCATATACATC

..(((((.((((......((((((.(((((((..(((((((((.(((((((..(.(((.....))))..)))).))).)))))))))..))))))).)))))).....)))).)))))....

GCGTACAGAGTAGTCAAGCATG*****************

miR160d-3p

GATTTGCTGTCATGCCTGGCTCCCTGTATGCCACAAGTGGATATCGATTTTGGCTGAAAAATTAGCTGCTGGTGGCGTACGAGGAGCCAAGCATGACCAGAGGAAG

......((((((((.((((((.((((((((((.(((((.....((((((......))))))..))))).)))))))))).))))))).)))))).)))......

CGTACGAGGAGCCAAGCATGA***

miR162a-5p

AAGCAAGAGTGACAGTTGCTGGAGGCAGCGGTTCATCGATCTCTTCCTGAGAATTTTTTTTTGGAAAGAAAGGAAGATAAAAATTAAAAGCATGAACAGATCGATAAACCTCTGCATCCAGCGGTTTACCTC
TTCGTCAAT

..(((((((.(..((..((((((.((((.((((.((((((((((((((.....(((((....)))))...)))))).......(((......)))..)))))))).)))).)))).))))))..))..))))
)).))....

********************GGAGGCAGCGGTTCATCGATC***
****

TCGATAAACCTCTGCATCCAG*********
****

miR162a

AAGCAAGAGTGACAGTTGCTGGAGGCAGCGGTTCATCGATCTCTTCCTGAGAATTTTTTTTTGGAAAGAAAGGAAGATAAAAATTAAAAGCATGAACAGATCGATAAACCTCTGCATCCAGCGGTTTACCTC
TTCGTCAAT

..(((((((.(..((..((((((.((((.((((.((((((((((((((.....(((((....)))))...)))))).......(((......)))..)))))))).)))).)))).))))))..))..))))
)).))....

*******************TGGAGGCAGCGGTTCATCGATC***
****

TCGATAAACCTCTGCATCCAG*********
****

miR162-5p

AAGCAAGAGTGACAGTTGCTGGAGGCAGCGGTTCATCGATCTCTTCCTGAGAATTTTTTTTTGGAAAGAAAGGAAGATAAAAATTAAAAGCATGAACAGATCGATAAACCTCTGCATCCAGCGGTTTACCTC
TTCGTCAAT

..(((((((.(..((..((((((.((((.((((.((((((((((((((.....(((((....)))))...)))))).......(((......)))..)))))))).)))).)))).))))))..))..))))
)).))....

*******************TGGAGGCAGCGGTTCATCGATC***
****

TCGATAAACCTCTGCATCCAG*********
****

miR162-3p

AAGCAAGAGTGACAGTTGCTGGAGGCAGCGGTTCATCGATCTCTTCCTGAGAATTTTTTTTTGGAAAGAAAGGAAGATAAAAATTAAAAGCATGAACAGATCGATAAACCTCTGCATCCAGCGGTTTACCT
CTTCGTCAAT

..(((((((.(..((..((((((.((((.((((.((((((((.(((.((...(((((((((((.....)))))))))))...........)).))).)))))))).)))).)))).))))))..))..)))
))).))....

********************GGAGGCAGCGGTTCATCGATC***
****

GATCGATAAACCTCTGCATCC*********
****

miR164a

GCGGGTGAGAACCTTCACGCTGGAGAAGCAGGGCACGTGCAAACCAACAAACAGGAAATTTGTTTCAATGTAATTTGCACGTGCTCCCCTCCTCCAACATGAGCTCCTCACCATTGAC

...((((((....((((.(.(((((.((..(((((((((((((...(((.....((((....))))..)))..)))))))))))))..)).))))).).))))...))))))......

********************TGGAGAAGCAGGGCACGTGCA***

CACGTGCTCCCCTCCTCCAAC*****************

miR164b-3p

AAACACTTGCTGGAGAAGCAGGGCACGTGCGAGCAGATGAGATCGATCTATAAATGTTGATCATATTTTCGCACGTGTTCTACTACTCCAACACGTGTCT

..((((.((.(((((.((.((((((((((((((..((((.(((((((.......))))))).))))))))))))))))))).)).))))).)).))))..

**********TGGAGAAGCAGGGCACGTGCG***

miR165a

TTCAGTTGAGGGGAATGTTGTCTGGACCGAGGATATTATACACACACACATACATATAAGTGTTGATACGTGATACAAGTTATCGATCATATATGTATATGTATTAGAGTATTCTCGGACCAGGCTTCATCC
CCCCTAACATGTT

..((((((.((((.(((..((((((.(((((((((((.......(((.(((((((((.(.(((((((..(......)..))))))).))))))))).))).....))))))))))).))))))..))).)
))).)))).))..

**TCGGACCAGGCTTCATCC
CCC**********

miR165a-5p

AAAGCTATTTCAGTTGAGGGGAATGTTGTCTGGATCGAGGATATTATATATACGCAGATACGTATATATATTAATGCAAGCAATTGATCATATATGTATATAGAGTATTCTCGGACCAGGCTTCATCCCCCC
TAACATGTTATTGCCTCTG

...((.((..((((((.((((.(((..((((((.((((((((((.(((((((.........((((((((((((......))))))).))))))))))))).))))))))))).))))))..))).)))).
))))).))..)).)).....

********************GAATGTTGTCTGGATCGAGGA***
****

miR166

CCAAAAGTTCAGGTGGATGATGCTTGGCTCGAGACCGTTCAATATGATCATTGTCATGATATGATGATAATGATGAGGATGATAATGATAATGATGTCGGACCAGGCTTCATTCCCCTCAACTTAAAC

....(((((.(((.((((((.((((((.((((.(.((((((...(.((((((((((((.....))))))))))).)...)))........))).).)))).)))))).)))))).))).)))))....

TCGGACCAGGCTTCATTCCCCT********

miR166a

AAGGGTTTTCTCTTTTGAGGGGAATGTTGTCTGGCTCGAGGACTCTGGCTCTATCTACTCATGTTGGATCGTCTTCGATCTAATCGAATCAAACTTCTATGTTTGAGATCTGATTAGGGTTTTAGCGTCGTC
GGACCAGGCTTCATTCCCCCCAATTATTGCTCCCTGAAT

.((((........(((.((((((((..((((((..(((.(((.(((((((((((.......(((((((((...))))))))).((.((((((.....))))))..)).)).))))))..))).))).))
)..))))))..)))))))).)))........))))....

********************GGAATGTTGTCTGGCTCGAGG***
****
**TC
GGACCAGGCTTCATTCCCC********************

miR166b-5p

TCTTTTGAGGGGACTGTTGTCTGGCTCGAGGACTCTTATTCTAACTCAATCTAATTTTATAAAATCTGCTTGATCTGATGATTGATTAGGGTTTTAATGTCTTCGGACCAGGCTTCATTCCCCCCAATTAT
....(((.(((((.((..((((((.((((((((....(((((((.((((((............................)))))))))))))......)))))))).))))))..)).))))).)))....
**********GGACTGTTGTCTGGCT***

miR166d-5p

AAGGGTTTTCTCTTTTGAGGGGAATGTTGTCTGGCTCGAGGACTCTGGCTCTATCTACTCATGTTGGATCGTCTTCGATCTAATCGAATCAAACTTCTATGTTTGAGATCTGATTAGGGTTTTAGCGTCGTC
GGACCAGGCTTCATTCCCCCCAATTATTGCTCCCTGAAT
.((((.........(((.((((((((..((((((..(((.(((.(((((((((((.......(((((((((....))))))))).((.((((((......))))))..)).)).))))))..))).))).))
)..))))))..)))))))).))).........))))....
********************GGAATGTTGTCTGGCTCGAGG***
****
**TC
GGACCAGGCTTCATTCCCC********************

miR166e

AAGGGTTTTCTCTTTTGAGGGGAATGTTGTCTGGCTCGAGGACTCTGGCTCTATCTACTCATGTTGGATCGTCTTCGATCTAATCGAATCAAACTTCTATGTTTGAGATCTGATTAGGGTTTTAGCGTCGTC
GGACCAGGCTTCATTCCCCCCAATTATTGCTCCCTGCTT
.((((.........(((.((((((((..((((((..(((.(((.(((((((((((.......(((((((((....))))))))).((.((((((......))))))..)).)).))))))..))).))).))
)..))))))..)))))))).))).........))))....
********************GGAATGTTGTCTGGCTCGAGG***
****
****
GGACCAGGCTTCATTCCCC********************

miR166g-5p

TCTTTTGAGGGGAATGTTGTTTGGCTCGAAGACTCTAGCTATTCATGTTAGATCTTCTTCGATCTAATCCAATCATATGTCCATGTTTGAGATCTGATTAGGGTTTTAGAGTCGTCGGACCAGGCTTCATTC
CCCCCAATTAT

....(((.((((((((..((((((.((((.((((((((..........((((((......))))))(((((((((...((((....)).))).))))).)))).)))))))).)))).))))))..)))))
))).)))....

*********GGAATGTTGTTTGGCTCGAAG**
****

miR166h-3p

AAGGGTTTTCTCTTTTGAGGGGAATGTTGTCTGGCTCGAGGACTCTGGCTCTATCTACTCATGTTGGATCGTCTTCGATCTAATCGAATCAAACTTCTATGTTTGAGATCTGATTAGGGTTTTAGCGTCGTC
GGACCAGGCTTCATTCCCCCCAATTATTGCTCCCTGCTT

.((((.........(((.((((((((..((((((..(((.(((.((((((((((......((((((((((....)))))))))).((.((((((......))))))..)).)).))))))..))).))).))
)..))))))..)))))))).))).........))))....

********************GGAATGTTGTCTGGCTCGAGG***
****

**TC
GGACCAGGCTTCATTCCC*********************

miR167

TCAAGCTGCCAGCATGATCTAAAAATCTCTGCATGTGGGGATTATCAGATCATGCTGCAGTTTAACCT

..((((((.((((((((((.(.((((((((....)))))))).).)))))))))))))))))).....

TCAAGCTGCCAGCATGATCTA***

miR167a

GACGGTGCACAGGCATCTGATGAAGCTGCCAGCATGATCTAATTAACTTTCTTTCTCCGTTTGTTTTCATGACGATGGAAAGAGATGAGTGTAGATTAGATCATGTTTGCAGTTTCACCCGTTGACTATCGC
ACCCTTA

...(((((...((((.(.(.((((((((.(((((((((((((.((((((((.((((((.(((.....))))))))).))))).))))...))))))))))))).))))))))).).).)).))...))
)))....

********************TGAAGCTGCCAGCATGATCTA***

****

GATCATGTTTGCAGTTTCACC***********

****

miR167a-3p

GGTGCACCGGCATCTGATGAAGCTGCCAGCATGATCTAATTAGCTTTCTTTATATCTGTTGTTGTGTTTCATAACGATGGTTAAGAGATGAGTCTCGATTAGATCATGTTCGTAGTTTCACCGTTGACTGTC

GCACC

(((((..(((((.(.(.(((((((((.(((((((((((((((((((((((((.((.((((((((((...))))))))))).))))))).))).)).)))))))))))))).))))))))).).).)).))).

.)))))

GATCATGTTCGTAGTTTCACC*******

******

miR167b

CTGCCCAAGGGAACGAGTGAAGCTGACAGCATGATCTATCTCCGAGTGATCAAACAAGAAACGCTGCGGCAGCCTCACTTCTTCCCGGCG

..(((...(((((.((((((.((((.((((.(((((...........))))).........))))...)))).)))))).)))))))).

*****************TGAAGCTGACAGCATGATCTA**

miR167d

TTTGAGAGGCTGAAGCTGCCAGCATGATCTGGTAATCGCTACATACGACATATATACATACACATGCTAATTTACTTCCACATATATCAATGTATCTCTCAAGTTAATATAATCTTAGTGTTCAAAAATGTG

TCTTCTCCTCTTTTTCATAATGAGTGATGTTACTATTTTAACTATTTCCCATATATATATATATATATATATATATATATATATATATATATATATATATATATATATATATATATATATTCATGCATGTATGTGT

GTGTATAAGTGAATAGAGATAGTGGTACCAGATCATCCTGCGGCCTCATCCACTCGAAT

((((((.((.(((.(((((.((.(((((((((((.((((((..(....(((.(((((((((((((((............((((.....)))).........((((((........(((((.(((....((((

...............)))).....)))...)))))..)))))).......((((((((((((((((((((((((((((((((...))))))))))))))))))))))))))))))))).......))))))))

))))))).))).....)..))))))))))))))))).))))))).))).)).)))))).

**********TGAAGCTGCCAGCATGATCT**

****

****

miR168

AGTCACCGTCGGGCTCGGATTCGCTTGGTGCAGGTCGGGACTAATTCGCTGACACAGCCACGTGGCTCTTTTTCTTATTGGTTTGTGAGCAGGGATTGGATCCCGCCTTGCATCAACTGAATCGGAGCTCCC

ACGGTGAAAA
..(((((((.((((((.((((((.((((((((((.((((((((((((.(((.((((((((...((........))...))).)))))..)))))))))).))))).)))))))))).)))))).)))).)).
)))))))...
********************TCGCTTGGTGCAGGTCGGGA**
****
CCCGCCTTGCATCAACTGAAT*******
****

miR168a

AGTCACCGTCGGGCTCGGATTCGCTTGGTGCAGGTCGGGACTAATTCGCTGACACAGCCACGTGGCTCTTTTTCTTATTGGTTTGTGAGCAGGGATTGGATCCCGCCTTGCATCAACTGAATCGGAGCTCCC
ACGGTGAAAA
..(((((((.((((((.((((((.((((((((((.((((((((((((.(((.((((((((...((........))...))).)))))..)))))))))).))))).)))))))))).)))))).)))).)).
)))))))...
********************TCGCTTGGTGCAGGTCGGGAC***
****
CCCGCCTTGCATCAACTGAAT*******
****

miR168b

AGTCACCGTCGGGCTCGGATTCGCTTGGTGCAGGTCGGGACTAATTCGCTGACACAGCCACGTGGCTCTTTTTCTTATTGGTTTGTGAGCAGGGATTGGATCCCGCCTTGCATCAACTGAATCGGAGCTCCC
ACGGTGAAAA
..(((((((.((((((.((((((.((((((((((.((((((((((((.(((.((((((((...((........))...))).)))))..)))))))))).))))).)))))))))).)))))).)))).)).
)))))))...
********************TCGCTTGGTGCAGGTCGGG***
****
CCCGCCTTGCATCAACTGAAT*******
****

miR168a-3p

AGTCACCGTCGGGCTCGGATTCGCTTGGTGCAGGTCGGGACTAATTCGCTGACACAGCCACGTGGCTCTTTTTCTTATTGGTTTGTGAGCAGGGATTGGATCCCGCCTTGCATCAACTGAATCGGAGCTCCC
ACGGTGAAAA
..(((((((.((((((.((((((.((((((((((.((((((((((((.(((.((((((((...((........))...))).)))))..)))))))))).))))).)))))))))).)))))).)))).)).
)))))))...
********************TCGCTTGGTGCAGGTCGGGAC***
****
CCCGCCTTGCATCAACTGAAT*******
****

miR169a

AAGTAGTGTGCAGCCAAGGATGACTTGCCGATTCGAAAAATATTTTAATACTTTATTAAGTCATCTTCTTAGTTTCAATTTTTTTGGAGAGCTAGGAAGAAATAACAATTTATTTCGTATTTAAAGAAAAAT
ATGATCGGCAAGTTTTCCTTGGCTACATGTTTCTTTGTGTCGCGCAGAGGTG
.......(((.(((((((((.((((((((((.................((((....))))..(((((((((((((((.....)))))..))))))))))(((((....)))))((((((((......))))
)))))))))))))).))))))))).)))...((((((((...))))))))..
********CAGCCAAGGATGACTTGCCGA***
****

miR169m

AGAATGATATTGAGCCAAAGATGACTTGCCGAATTTACCAACTAATCTAAAACTGATAGTGGTCATTGGCAAGTTGACTTTGGCTCTGTTTCATTCTC
(((((((.((.(((((((((..((((((((((....((((.(((.((.......)))))))))..))))))))))..))))))))).)).))))))).
**********TGAGCCAAAGATGACTTGCCG***

miR171b-5p

GTTGATGTAAAGGTAACGCGAGATATTAGTGCGGTTCAATCAAATAGTCGTACTCTTAGCTATTAGAGATCGGTTTTGTTCGATTGAGCCGTGCCAATATCACGCATAAAACCAACATGTCGA
.(((((((...(((...(((.((((((.(((((((((((((.((((((((..((((........))))..)))..))))).))))))))))))).)))))).))).....)))...)))))))
********************AGATATTAGTGCGGTTCAATC**

miR172a

GGTACTTGCAGATGCAGCATCATTAAGATTCACAAGAGATGTAGTTTCTTTTGTTTTCACCTCTCTATTCGAAAACAAAGGGGTCATCTCGAATGAGAATCTTGATGATGCTGCATCAGCATATAC
.(((..(((.((((((((((((((((((((.((.((((((....((((((((((((((............)))))))))))))).))))))...)).)))))))))))))))))))).)))..)))

AGAATCTTGATGATGCTGCAT********

miR172a-5p

CACGGTCGTTCTTTGCTGCTGTGGCATCATCAAGATTCACATCGCTAGATGAACGGTGGTTATTCACTCTTCCCAAAGTTCTCTGATGAAAATGAGAATCTTGATGATGCTGCATCGGCAATCAACGGCTAT
TCT

...(((((((..((((((.((((((((((((((((((.(((((.((((.((((..(((...............)))..)))))))).)))...)).)))))))))))))))))).))))))..)))))))..
...

********************GTGGCATCATCAAGATTCACA***
***

miR172b-5p

ATCAGTCGTTTTCTGTAGGTGCAGCACCATTAAGATTCACATGGAAATTGAAAAACGCCTAATTTATATTAATTAGGGTTTTGATATGTGTATGAGAATCTTGATGATGCTGCATCAACAACCAACGGCTAC
CAA

...(((((((...(((.(((((((((.(((((((((((.((((.(......(((((.(((((((......))))))))))))......).)))).))))))))))).))))))))).)))...)))))))..
...

********************GCAGCACCATTAAGATTCACA***
***

miR172c

GTGATTGCAGTCATTTTTTGCTATTGCATCATCATCAAGATTCAGAAATCATATTCTCTGATGGGTTCTTCGTTCGAGCCTGTCTTTTTCTGGTTTGAGAATCTTGATGATGCTGCAGCGGCAATCAA

(((((....)))))...(((((.(((((.((((((((((((((..((((((........(((((((((.......)))))))))......))))))..)))))))))))))).))))).)))))....

AGAATCTTGATGATGCTGCAG*******

miR172e

GGTTTTTGCAGATGCTGCATCATTAAGATTCACAAGAGATGTGGTTTCCTTTGATTATCGCCTCTTGGGAATCTTGATGATGCTGCATCAGCAAATAC

.((.(((((.(((((.(((((((((((((((.((((((..(((((.((....))..))))))))))).))))))))))))))).))))).))))).))

GGAATCTTGATGATGCTGCAT*******

miR390a

GTAGCAGAGAAGTAGCTGTAAAGCTCAGGAGGGATAGCGCCATCACTCACCCTTACTCTATATATGTCATTGTATAAGTAATGGCGCTATCCATCCTGAGTTCCATGGCTTCTTCTTGCTTTTAT

..((((.(((((.((((((..(((((((((.((((((((((((..........((((.((((((......))))))))))))))))))))))).)))))))))..)))))).)))))))))).....

********************AAGCTCAGGAGGGATAGCGCC**

miR390b-5p

AGAATCTGTTTAGCTCAGGAGGGATAGACACGTGATACTGATGATTATATGTTCATAGTTGTATACGTACTTGGCGCTATCTATCTTGAGCTTCATGGCTTCT

((((.((((.((((((((((.(((((((.(((.(.(((..(..(((((......)))))..)....)))).))).))))))).)))))))))).)))).))))

*********TTAGCTCAGGAGGGATAGACA***

miR391

TAAGATTTGCTTCGCAGGAGAGATAGCGCCATCACCTATCTTCTGATAGTCTAGCTGATGGTGATGACGGTATCTCCCCTACGTAGCAATCCTTAT

((((..(((((.((.(((.((((((.((.(((((((.(((..(((......)))..)))))))))).)).)))))).))).)).))))).)))).

**********TTCGCAGGAGAGATAGCGCCA***

miR393a

CATAGCAACTAGAGGAAGGATCCAAAGGGATCGCATTGATCCTAATTAAGGTGAATTCTCCCGATTATTTTATTGGTTATTGGCAAAAGAAATACCAAAAAATTATTGGTTAGTTTTGGATCATGCTATCCC

TTTGAATTCATCCTTCGGTAGCTTTCAT

...(((.(((.(((((.((((.(((((((((.((((.(((((.((((((..........(((((.(((((.(((((.(((..........))))))))))))).)))))))))))..))))))))).)))))

)))).)))).))))).))).))).....

********************TCCAAAGGGATCGCATTGATCC**

****

miR395b

AATGCCACCTTGAGTTCCCTTTAACGCTTCATTGTTCAAAACTGAGTTGTATCATATCTACAAGATAAATCAATCAAATGAACACTGAAGTGTTTGGGGGGACTCTAGGTGGCAT

.(((((((((.((((((((((.(((((((((.((((((....(((.(((.....(((((...)))))...))))))..)))))).))))))))).)))))))))).)))))))))

CTGAAGTGTTTGGGGGGACTC********

miR396a

CTCTGTATTCTTCCACAGCTTTCTTGAACTGCAACCTTCTTCAGATTAGTTTTTATTCTCGTATATATATATTGCCATAGCATAGTTATGTTAATTTAAACTGCATCTGTTCGATTGATATGCGGTTCAATA

AAGCTGTGGGAAGATACAGAC

.((((((((.((((((((((((.((((((((((.(..((..(((((((((((..(((..((((..((.....(((....)))))..))))..)))..)))))).)))))...))..)...)))))))))).)

))))))))))).)))))))).

*****TTCCACAGCTTTCTTGAACTG**
****

miR396b

TTTTTCCACAGCTTTCTTGAACTTCCTTTTTCATTTCCCTTCTTTTAAAGATGTAATAACTAAAGATTGTTTTTAAACATTTAACACTCTAGAAACAAAAAAAAAAGCTCAAGAAAGCTGTGGGAAAACATG
TCAGGTTCTTTTAAA

((((((((((((((((((((.(((..(((((..((((.......(((((....(((.(((........))).)))....)))))).......))))..)))))..))).))))))))))))))))))))....
....

***TTCCACAGCTTTCTTGAACTT**
****

GCTCAAGAAAGCTGTGGGAAA***
****

miR396b-3p

TTTTTCCACAGCTTTCTTGAACTTCCTTTTTCATTTCCCTTCTTTTAAAGATGTAATAACTAAAGATTGTTTTTAAACATTTAACACTCTAGAAACAAAAAAAAAAGCTCAAGAAAGCTGTGGGAAAACATG
TCAGGTTCTTTTAAA

((((((((((((((((((((.(((..(((((..((((.......(((((....(((.(((........))).)))....)))))).......))))..)))))..))).))))))))))))))))))))....
....

***TTCCACAGCTTTCTTGAACTT**
****

GCTCAAGAAAGCTGTGGGAAA***
****

miR397a

TGGATGAACATCATTGAGTGCAGCGTTGATGTATCTTTCTTCTTTCTCCTAATGTTGGATGGGTAAAAAGAACTTACACCAGCGTTGCGCTCAATCATGCTTTTCTAA

((((.((.(((.((((((((((((((((.(((((((((...(((..(((.......))).)))...)))))...)))).)))))))))))))))).))).)).)))).

**********TCATTGAGTGCAGCGTTGATGT**

miR398a-5p

AAATCTCAAAGGAGTGTCATGAGAACACAGATCCTCTTGTTTCTTTAAGATTTCCACTGAAACCATTGAGTTTTGTGTTCTCAGGTCACCCCTTTGAATCTC

.....(((((((.(((.(.((((((((((((..(((..(((((...............)))))....))).))))))))))))).).))).))))))).....

**********GGAGTGTCATGAGAACACAGA***

miR398c-5p

GATCTCGACAGGGTCGATATGAGAACACATGTGCAGTCAACGGCTGTAATGACGCCACGTCATTAGAACAAATCTTCCTTTCCATGTGTTCTCAGGTCACCCCTGCTGAGCTCT

((.((((.(((((..(((.((((((((((((.(((((.....)))))(((((((...)))))))..................)))))))))))).)))..))))).)))).)).

**********GGGTCGATATGAGAACACATG***

TGTGTTCTCAGGTCACCCCTG*******

miR399b

TCACTAGTTTTAGGGCGCCTCTCCATTGGCAGGTCCTTTACTTCCAAATATACACATACATATATGAATATCGAAAATTTCCGATGATCGATTTATAAATGACCTGCCAAAGGAGAGTTGCCCTGAAACTGG
TTC

..(((((((((((((((.((((((.((((((((((.((((.......(((((........)))))((.(((((........))))).))......)))).)))))))))).)))))).))))))))))))))
)..

TGCCAAAGGAGAGTTGCCCTG*****
***

miR399d

TGACTGGATTACTGGGCGAACACTCCTATGGCAGATGGTTTTGGCTTGCTTGAGATGCAAGCAAGATGTTTCTCTGCCAAAGGAGATTTGCCCCGCAATTCATCC

.((.((((((.(.(((((((..(((((.(((((((.((......((((((((.....)))))))).....))))))))).))))).))))))).).)))))))).

TGCCAAAGGAGATTTGCCCCG********

miR399d-5p

CAATGTATGGCTGGATTACTGGGCGGATACTCCTGTGGCAGATGTTTTTAGCATTGCTTGAATTGCAAGCAAAATGCTTCTCTGCCTCTGCCAAAGGAGATTTGCCCCGCAATTCATTCCATACAAAAC

...(((((((.((((((.(.((((((((.(((((.(((((((.((....(((((((((((.....))))))..))))).....)).))))))).))))))))))))).).))))))..)))))))....

********************GGGCGGATACTCCTGTGGCAGA***

miR399g

ATATAAGCTCACTAGTTCCAGGGCTTCTCTCTATTGGCAGGTCCCTTATTTTCACACACATACACAAACACACACACACACACATATATATATATATATATATTCATGTTATTAGCAGATTTTCACATATAT
ACACACCCACACAAACATATATTCCTGTTATATCAGAAAGTCAAAATGATCTGCCAAAGGAGAGTTGCCCTGCAACTAGTTTTAGCTTAGAT

...((((((.(((((((.((((((..((((((.((((((((((......................................((((..((((((....))))))..))))........(((((((..(((((.
(((.....................))))))))..))))))).....)))))))))).))))))..)))))).)))))))...))))))...
*********************GGGCTTCTCTCTATTGGCAGG**
****

miR399h-5p

AAAGGTGAAAATGCATTACAGGGCAAGATCTCTATTGGCAGGAAACCATTACTTAGATCTTTGCATCTCTCTGTGCATTGATTTGTAGTGAGTTCTCTGCCAAAGGAGATTTGCCCGGTAATTCTTCTGCAC
CAATAT
...((((.....(.(((((.(((((.(((((((.((((((((.(((((((((..(((((..(((((......)))))..))))))))))).))).)))))))).))))))))))))).))))).).....)))
).....
********************GGGCAAGATCTCTATTGGCAGG**
******

miR408-5p

ACGAGAGAGAGACAGGGAACAAGCAGAGCATGGATTGAGTTTTACCAAAACATCAAATGACTATGTTTGGTCTCTTCCCATGCACTGCCTCTTCCCTGGCTCCCTCTTTC
..(((((.(((.(((((((...((((.((((((.((((((((.....)))).))))..(((((....))))).....)))))).))))...))))))).))).)))))..
***********ACAGGGAACAAGCAGAGCATG**
ATGCACTGCCTCTTCCCTGGC*******

Non-Conserved miRNAs

miR1885a

GTTCAAAGAAAAAGAATCATACTTTCATTGACAATATTGATAATGAAATCAATAGAGAATGATAATGAGCTCTTACATGCAATTCAAAAATCTAGTATCTCTTAGATCTTCTAATTGCTTGTAAGAGTTGAT
GACCAATGGGTTATCCTCTATTGATCTCATCATCAATGAAAGGTATGATTCCCTCTCTTTGAAA
.((((((((....((((((((((((((((((......(((...(((.((((((((((.........((((((((((.((((((..((.((((((......)))))).))..)))))).))))))))))(((
((((....))))))))))))))))))).)))))))))))))))).)))))))))....)))))))).
****
********************************TCAATGAAAGGTATGATTCCCT**********

miR1885b

TTGTCTTCGCGAGCTTCCTCTAGAAGATGTGTGAAGAGGCTTTCTAAATCACACTCATAAAGAGTTCAAAGAAAAAGAATCATACTTTCATTGACAATATTGATAATGAAATCAATAGAGAATGATAATGAG
CTCTTACATGCAATTCAAAAATCTAGTATCTCTTAGATCTTCTAATTGCTTGTAAGAGTTGATGACCAATGGGTTATCCTCTATTGATCTCATCATCAATGAAAGGTATGATTCCCTCTCTTTGAAATTCCT
TGTGAATGTGACTGAAAAAAACCCTTACATACATCTTCTCCGCGGAAGCTCATGAGAACAA
.(((..(((.((((((((.(.(((((((((((((((.((.((((....(((((.((((((.(((((((((((....((((((((((((((((((......(((...(((.((((((((((..........((
((((((((.((((((..((.((((((......)))))).))..)))))).))))))))))(((((((....))))))))))))))))).))))))))))))))).)))))))))....)))))).))))).)
))))).)))))..))))....))))).))))))))))))..).)))))))).)))..))).
****
****
*****************************TACATCTTCTCCGCGGAAGCTC**********

miR1863

TCTTGCCATGACAGAGATTATCTCCTGATCTGCAAAACAAATACCTAGTGTAGGATCTACGTGGGCTCAAGGCTTCTTCTTGAGCTTCTTCACGTCCATGGCTCTGATACCATGTAGATTTTAGGGTAAAAA
TGAAAACAATA
..((((...(((((((.....)).))).)).))))......(((((....((((((((((((((..(((..(((....(.((((....)))).).....)))..)))..))))).))))))))))))))...
....
TGGCTCTGATACCATGTAGAT***********
****

miR2111a-3p

CGATGATGAGTATTGGTGAGGACCGGGTAATCTGCATCCTGAGGTTTAAAGCTTAATTTACGCAGGAAATTTGTATACGCATATACGTATGTGTATTAGTATACCTTTTAGACCTCAGGATGCGGATTACCT
CGTTCTTACTTACAATACACGTACTCG
(((..(((.((((((((((((((.((((((((((((((((((((.(((((((.........)).........((((((..((((((....))))))..))))))..))))).)))))))))))))))))))
.))))))))...)))))).)))..)))
**GACCTCAGGATGCGGATTACC*
****

miR2111c

GAGTATTGGTGAGGACCGGGTAATCTGCATCCTGGGGTTTAAGGTTTAACTACACACAAAGTATGCGTACGCGTATATATGTGTGCGTGCGCGTGTGTTAGTATAGCTTTTAGACCTCAGGATGCGGATTAC
CTCTTCCTTACAATACGTACA

..((((((.((((((..(((((((((((((((((((((((((((((..(((((((((.......((((((((((((....))))))))))))))))))).))))..))).)))))))))))))))))))))))
))..))))))))))))).....
********************TAATCTGCATCCTGGGGTTTA***
****

miR394a

AGAGTTTCTTACAGAGTTCTTTGGCATTCTGTCCACCTCCTTTCTGTATATATTTATGCGTATAAGTGTTTGTAACTACGTTGTGTTTTGTGAGAGGAGGTGGGCATACTGCCAATAGAGATCTGTTAGGGT
TTCTTT
((((.((((.(((((.((((((((((...((((((((((((((...(((((.....((((...(((.......))))))))))).....))))))))))))))...)))))).)))).))))).)))).
))))..
*****************TTGGCATTCTGTCCACCTCC***
******

miR394a-3p

GGCGCTTACTGAAGAGTTCCTTGGCATTCTGTCCACCTCCTCTCGCCGATCTTGCAGAAAGTTTACCAGTTCTGTTGCATTTTTTTTGGAGGTGGGCATACTGCCAATAGAGCTGTGTAGGCCTCC
((.((((((...(.((((((((((((.(.((((((((((............((((((.(....)...))))))............))))))))))).).)))))).)))))).)))))))))..
AGGTGGGCATACTGCCAATAG*************

miR394b-3p

TGAAAGATGGAGGTGGTCATACTGTCAACAGACATCTGTTAGGGTTTCTTTGTAAAACTCTCTTGATATACCATATTTATCAATGTTTTGATTTTGGTGAGCTTGATCTGTTATTTGGTGTTGACAAGCCTC
CACTTGGCAT
((.(((.(((((((.(((((((((..((((((.(((....(((((((.......)))))))...))).(((((.....(((((....)))))..))))).......))))))...))))).))))..)))))
)))))..)).
**********AGGTGGTCATACTGTCAACA**
****

miR400

AAAGTTGCTGAAGATTGTTTTATGAGAGTATTATAAGTCACTGCGTTTGGTAATCTGAGGTGTTGCTTTCCTAAACGAAGTGACTTATAATGATCTCATGAATCGATTTTTGCAAAGTCTTG
....((((.((((((((.(((((((((.((((((((((((((.(((((((......(((......)))..))))))).)))))))))))))).))))))))).))))))))))))).......
*********************TATGAGAGTATTATAAGTCAC**

miR400-3p

AAAGTTGCTGAAGATTGTTTTATGAGAGTATTATAAGTCACTGCGTTTGGTAATCTGAGGTGTTGCTTTCCTAAACGAAGTGACTTATAATGATCTCATGAATCGATTTTTGCAAAGTCTTG

....((((.((((((((.(((((((((.((((((((((((((.(((((((......(((......)))..))))))).))))))))))))))).))))))))).)))))))))))).......

********************TATGAGAGTATTATAAGTCAC**GACTTATAATGATCTCATGAA********************

miR403

GAGTCATATTACATGTTTTGTGCGTGAATCTAATTCATATGCTCATGTAATAATCCTATCTTGTTGTTGGATTAGATTCACGCACAAACTCGTAATCTGTCT

.((.((.(((((..((((.((((((((((((((((((...((....................))...))))))))))))))))))))))..))))).)).))

TTAGATTCACGCACAAACTCG*******

miR535b

GATGTTGTGTGACGACCGAGAGAGCAAGACTTGTCATCACCCATGAACGGATCATGGTTGTTTACGGGCGTGCTCTCTCTTCTTGTCATACGGTC

....((((((((((((((((((((((((((((((...((.((((((.....)))))).))...))))))))))))))))))))))))))))))..

*********TGACGACCGAGAGAGCAAGAC***

miR5161

TTCTAGAATATACTACCTCTGTTCCAAAATATAAGCATTTCCAGGATTCAAATCTTGTTCCACAATATAAGCATTTCTACCACTATTTACACCACTACTTGTCCCATCAATCACTTTCCATCCAATTTTTTC

CCCATCCAACCCCCAACCCCCAACCCCCCTAACTCTCCCTCATTTATTGAGGGGCACCAAGTCTTTTTCCTCAACCTTAATCTTTGCTAAACAACCTAGAAATGCTTATATTCTGGTCTGGATAGAAGGTA

GGATAGTTCACT

..(((..(((((((.((((((.((((.(((((((((((((.(((.........(((.....)))....((((........................(((......))).......................

...................................(((((((.....)))))))................................))))......))).))))))))))))).)))).)))))).))))))

)..)))......

****

***TCTGGATAGAAGGTA

GGATA*******

miR5227

TTAAAGAAAATGAAGATGAAGAAGTTGAGCAGTGTCACCTATTTCTCCTACTTCGCTGAAATTCTTGCCATATGTCATGGTCTTCCTTATCATCTCTTCTGACGCGTTGATTCATCTCATCATCTCTTTTTT

TTTGC

.(((((((((.((.(((((.((.(.((((((((((((...(((((..(......)..)))))....(((((.....)))))..................))))))..)).)))).))).))))))).)))))
)))).
**********TGAAGATGAAGAAGTTGAGCA***
*****

miR6273

CTGCAAACCAAAAAAAAAAAAAAAAAATCATCCAGCATTCCTTTTACAGCTATTTACTGATTCAGTAAGTAGTACCATAAGGAAAGGGCTGATTTTTTTTTTTTGCTTGTTGATTTGTTAAAT
..(((((.(((......((((((((((((((.(.(((((((((.....(((((((((((...))))))))))).....))))))))).).))))))))))))))).....))).))))).....
AAAGGGCTGATTTTTTTTT********************

miR6284

GTCCTGTTGAGAAGCTCTACATTTATAGGTCCGTCGCTGGTCCAAGGTTTGACATGCCTGGCTTTGTTTATGACCAGGATGAAGCTTCTGCAGTGAG
.((((((..((((..(((.(((((((.((((((.....((.(((.(((.......)))))))).....))))))))))))).))))))))))).)).
TATGACCAGGATGAAGCTT********

miR824

TATCACCATTTGTACTTGAGTTGTCTCTCATGTCTAGACCATTTGTGAGAAGGGAGTTTTTGTTTACACCAATACCCCCCAGTCTCTAAATTTGTAAGAAGTATTATGCTCAATTAAGGAACATAGCTAGTT
CGACATACCATACTACCCTTTTAAAACTATCCTATATGTTTGATGCTAGCATAGCGTAATTTTGTGTTCTCATGGTGCAGCAGGGTTAGTTTATTGTGTACCTCTAGATATATATTCTCTTGCTGCGTAGG
GTTCCCAAGCTGCCAAAAACTTTTAAAAATTAGTGATCTGTTCCCCAAACCCCCATTCAATAAGAAAGGTCTACCTGTAGCTCACAGTCACAGCTATTAGAGCTGTCCTTGCTTCTTTGGGTTTACTGTTTT
AGTTAATTAGTTACCCTATGAAAGTCTGATCCTTCAGAAGTTAGGTATAGAAGTAAGTCGATTAAGTTCATTCATGACTTTTCCAGATGTTGCATACATAACTTTTTTTATTGTATTCGAACTAGTCCAAG
CCGATTCTCAAAATCATATAAAATCATTTACACCTTGGTCATGGTAGCTAAGAATATTGTATCTAAAATTGGGGAGTGGGGAGATGTTTGGTTATATTCCCTTCTCATCGATGGTCTAGATGTGCGAGGTGA
CTCTCATGGAGGTAAAGAACAATGGTGAT
.((((((((.((.(((((((..((((.((((((((((((((.(((((((((((((.........(((((((.((((((.(((((((.((((.....((((((..((((.(((((((......(((((((
(((.((((..(((((...(((((((((......((((......((((((...))))))).....)))).......(((((((((((...((..(((.((((........)))))))..))))))))))...((
....))......))))......))))))))).))))).....................((((((((((((.....((((((((......((((((.....)))))).(((((((((..((((..((((....
.......)))).)))))..(((...(((((....)))))..))).....)))))))))..((..(((((.......))))).))..)).))))))......))))))))))))).)))))))))))))).....
..((((.....))))................)))))))....).)))....))))))......)))).))))))).))))).).)).)))))...))))))))))))).))))))))))))))).))))..)
)))...........))).)))))))))))

***TAGACCATTTGTGAGAAGGGA**

****

****

****

****

****

miR824-3p

TAATTTGGGAAGTGGGGGAGAGGGGGGGGGAGGTGTTGTATTATTCCCTTCTCATTCGATGGTCTAGATGTGCGAGGT

..(((((((..((.(((((((((((((.((.........)).)))))))))).))).))..)))))))..........

CCTTCTCATTCGATGGTCTAGA********

miR860

TCAAGTGTAATGTATGTAGTCCAATCTATTGAAGTACTAGCACACCATCTCTAGTGAGGTGATGTGGTTTGAGTAGTTCAATACATTGGACTACATATATTACATTTG

.((((((((((((((((((((((((.(((((((.(((((.((((.((((((....)))))).)))).)...)))).))))))).))))))))))))))))))))))))

*************ATGTAGTCCAATCTATTGAAG**

TCAATACATTGGACTACATAT*******

Novel miRNAs

rsa-miRn1

ATGAGATAGAAGAAGAGGAAGAGGATGAAGATTATGGAAGGACAAGCCTGGAGACTGTGCCAGAGAGCCCGTTTGAAGGAACCAGTGGAACAGTGTCTTCGAGCGCAGAAGACGACGTATATGTTGGAGTTG

GTAAAGGAGACTCAAGCATTGTGGCATTGCGTTGGGCTCTCGACAATCTAATCCCTTCTTCTTCTTCTACACTTGTT

(..((.(((((((((((((((.((((..(((((......((....((..(....).)).)).(((((((((((((((((...((.((...)).)))))))))))).........((((((.((((((.(((.(

.(..((....))..).)))).)))))))))))))))))))))))))))))...))))).))))))))))..))..).

**********AGAAGAGGAAGAGGATGAAGAT**

****

rsa-miRn2

TGGCTGGCTAGAGATCTCTTCGATCTCGATCTCCTCTTCAGAATCAGCAAGGGAGGGTGATTTGTTGCCCGAGGAAGAGGGGAGGATGGGTGGGTTTCTCCAGCGC

..(((((..((((((((..((.((((...((((((((((....((.((((.(((......))).))))..)).)))))))))))))).)).))))))))))))))..

AGGGGAGGATGGGTGGGTTTC*****

rsa-miRn3

GCTAACTCCACTACAAAAGTGTACTGCGGCAATTCGTATGTTAGCCTATGGTTGTGCGGGTGACATGGTGGACGAATATCTCCGCATGGGTGAGTCCACTGCGTTATTGTGTTTGGAAAAATTTACTCAAGG

AATCATTGATTTATATGGAGAGAAGTATCTACGAAAACCCACACCTGAAGATCTTCAACGACTACTTGATATCGGAGAGGTACGCGGTTTCCCTGGGATGATAGGAAGCATTGACTGTATGCATTGGGAGTG

GAAAAATT

......((((((.(...((((((.(((((((((.(...(((((.((((.((..(((.((((....(.(((((......((((((..((((((((((((..(((......))).))))....))))))))..(

((((...)))))...))))))......))))).)..)))).)))(((.....((((..(((..........)))..))))....)))...)).)))).)))))....).)))).)))))))))))..)))))

))......

****

CATTGACTGTATGCATTGGGAG

****

************ACAAAAGTGTACTGCGGCAATTCG**

****

****

rsa-miRn4

GGCGGTCGTCGGTATATTCCGACGACAATTCCGACGAAAGCCCGACAAAAATTATAACCGTTACGGTCGTCGGAATTCCGTCGGAATATACCGACGCCGT

.((((.((((((((((((((((((..(((((((((((....(((...................))))))))))))))).))))))))))))))))))))))

TCGGAATTCCGTCGGAATATA*******

**************TATTCCGACGACAATTCCGACG**

rsa-miRn5

CTCATCGTAGGAGAAAACGCTTCCATATCTAGCAGTAGGTTATTGGTTATTGGTGTCACCCTTGACCAGATCAGTTGGTGACATTTTATTATGTGGGAGAGTTGTTAAAAGGTGAGATATTTGATTCGAGGG

ATGCCAAGCACACCAATAACCTACCGATAGATGTGGAAGCGTTTCCGGTGAA

.(((((...(((...((((((((((((((((.(.((((((((((((..(((((((..((((((.(((.((((((.((..(((((((((.........)))).)))))..)).)))....))))..)))))))

)))))))....))))))))))))).).))))))))))))))))))))))))).

******************GCTTCCATATCTAGCAGTAGG***

****

****

*********************TACCGATAGATGTGGAAGCGT**********

rsa-miRn6

CCCATGATGATTTGCGTGAGTATGTGGATGTGTATCTGCATGTCCGCCACATCTGTTACTCATGAAAATCATCATACACACGAAAATCACATCCACATACTCACGAAAATCATATGGGA

((((((.((((((.((((((((((((((((((.....((......))....(((((.....(((.....))).....))).))....)))))))))))))))))).)))))))))))).

**********TTTGCGTGAGTATGTGGATGT**

ATCCACATACTCACGAAAATC******

rsa-miRn7

AGAGCTCTTCCCATCCCGTTCAGTTCTCCTTTTGCTTCACATTGGATCAGTCTCGTCAAGACTGGTTCGATACGAAGCAAACGAGAATTGAACGGAACAAAGCACA

...(((.........(((((((((((((..((((((((..(((((((((((((.....)))))))))))))..)))))))).))))))))))))).....)))...

****************CGTTCAGTTCTCCTTTTGCTTC**

AGCAAACGAGAATTGAACGGA*******

rsa-miRn8

AACTGTGAGTTTCAGAACGATATAAAAGATCATGGATACAGTAAAGAGATTTAACTCAAATGAGGCTAGTATATCTATGGCCTTTATATCGTATTCGAAATTGACAATT

...(((.((((((..(((((((((((.(..((((((((...((((....)))).(((....))).......))))))))..))))))))))).)..)))))).)))...

************CAGAACGATATAAAAGATCATGG**

ATGGCCTTTATATCGTATTCGAA********

rsa-miRn9

AGGCGTGACGTTCGAATCCCTTTCATGTGTCGTGACATCCCAGAAAAACTTCATCTAGACATGTGGGATGTGATTGTCAAGTT

.(((.(((((.(((.(((((...(((((.(.((((...............))))..).))))).))))).))).))))).)))

ATGTGGGATGTGATTGTCAAG

rsa-miRn10

TGCAACTCCGGTGACCGGCGCGTGGCGGCTCCCTTAGCGCCACCGCCGGTACCGTTAGCCTAGCTTTCGTTGTG

.(((((..(((((.((((((.((((((.((.....)))))))))))))))))))..(((...)))...))))).

**********GTGACCGGCGCGTGGCGGCTC***

rsa-miRn11

ATATATGTATATGCCTGGCTCCCTGTATGCCATACACTAAGTTCATCGACCATCGGCGACCTCGGTGGATGGCGTATGAGGAGCCAAGCATATTCTCAGCATAGAC

...(((((((((((.(((((((..(((((((((.(((..((.((..((.....))..)).))..))).)))))))))..))))))).)))))).....)))))...

**********ATGCCTGGCTCCCTGTATGCC***

rsa-miRn12

ACTGGATGGAATTTCAGTGAATTAGACTGAGAAGAATCTTGAAGTCCTAGCTTTTTGTTCGATACAAAAAAGTAAAGTATGTAGGTCTAAAATTTTTAGCCTGTTCTCCTTTGGTAGTTTGACCGCGAAATT
TTTTTTCTGCA

...(((.((((((((.(((..((((((((.((((...............(((((((((.....)).))))))).........((((.((((.....))))))))......))))..)))))))).)))))))))
))...)))...

**GGTAGTTTGACCGCGAAATT
T**********

rsa-miRn13

AGGGTTAAGGGTCTGTATGGTATGGGTGGAGGAGGTAAAATCCTCAATTCACATTTGATAATGATGTGATTGTGCCTACTCAGCCCATACATACAGACACTTAATCCCTG

((((((((.(((((((((.(((((((.(((.(((((.......((((.((((((.......)))))))))))))))).))).)))))))))))))))).))))).)))).

**********GTCTGTATGGTATGGGTGGAGG**

rsa-miRn14

AAAAAAAAAGGCTAATGAGATCGAAATACTGAATCATAAATAGAGTTCCATTTCGAATTGGCTAGATAAAACAAAGTCTTGCCTATTATGACAAATAAATCAAAGACTTTCCAAAAAAATTTTTTTTATTCA
TATATTTTTTATTTTAAAACTAGGTTTCGGTTCGGTTAGTTGAGCTTGAA

.........(((((((..(((((((((.(((......((((((((..............(((.((((........)))).)))...(((((...(((((..(((((.(((.....))).))))))))))))))
))....))))))))......))))))))))))..))))))))).........

**********GCTAATGAGATCGAAATACTGA**
****

****
*********************AGGTTTCGGTTCGGTTAGTTG********

rsa-miRn15

TTGGAGAAGACAGAGACAGAGAGGAGAAAGGAATCGTGTAAGCAAGCACAAAACTTTCCTTTCTTCTATCTCTATCTCTCTCCTTTCGAT

...((((.((.(((((.((((((((((((((((..((((......))))......)))))))))))..))))).))))).)).))))...

**********CAGAGACAGAGAGGAGAAAGGAA***

rsa-miRn16

TAAATACTCTATATACTGAAGTTTATACTCTTCAATCTAGTTAAAAAATTTCAAACCACATTGTACATTTGAATTTATGTACTCTAAACTATCTTTCATGTTATATAGGAATTGTAATAATTTTTTAATATT

TATTCTACTAAAGTTTCATATATTGACTAGATTAGTGGAATTAGGATCATAAAATCTTCATTATCTAGAGAAATGA

......(((((.(((.((((((((((..((((.......(((((((((((.(((.((.....((((((........))))))....(((..........))).....))..)))....)))))))))))...

.((((((((((((((.........))))...)))))))))).))))..)))))..))))).))).)))))......

**********ATATACTGAAGTTTATACTCT***

****

rsa-miRn17

AGAGGGTAGAAATATCTGCCGACTCATCCATCCAAACACTCATGGTTATGAAACAAGAAATGTTAAACCACAGTGACTGTGTATGAATGATGCGGGAGATGTTTTTCATCTC

.((((..((((((((((.(((..((((.(((.((..((((..(((((....((((.....)))).))))).))))....)).))).))))..))).))))))))))..))))

*************ATCTGCCGACTCATCCATCCA**

TATGAATGATGCGGGAGATGT*******

rsa-miRn18

CTCGCCAAGCAGGTCGGCGTCCCCGACATGGTCGTCTTCCTCAACAAAGAGGACCAGGTCGACGACGCCGAGCTGCTCGA

.(((...(((((.((((((((..((((.(((((.((((........)))).))))).))))..)))))))).))))))))

****************GCGTCCCCGACATGGTCGTCT***

AGGACCAGGTCGACGACGCCG*******

rsa-miRn19

TAATATTTTTTGAGCGGCAACTATTGTAGGTTTCAAACTATATTTGACCCTATATTAATACTGATCCTTATAGTTTGAAATCTACAATAGTTGCAGCTGAGAAGATATTAT

((((((((((.(((.(((((((((((((((((((((((((((...(((..(((....)))..).))..))))))))))))))))))))))))))).))).)))))))))).

**********TGAGCGGCAACTATTGTAGGT**

rsa-miRn20

ATTGATGCGTTTGACGTTTCTCGAACTCAAGACCTCAATTGGTGACAACTTGTCCAGAGTTGTTAAAATGGTGAGGTCTTGAGTTCGAGGGACGCCAAGCGCACCGA

.(((.((((((((.(((..((((((((((((((((((.....(((((((((.....)))))))))......))))))))))))))))))..))).)))))))).)))

*************ACGTTTCTCGAACTCAAGACC***

TCTTGAGTTCGAGGGACGCCA********

rsa-miRn21

GGTTGGGATCGCTTGTTGGAGTCGTTGCCAGATGGTTGGGTGTATTGCGATGCTGATGGCTCACAGTTTGACAGCTCGCTATCACCATACTTGATCAATGCAGTACTCAACATCCGCTTAGAATTCATGGAA

GAGTGGGACGTAGGGGAGGTAATGCTGAGAAATTTGTACACCGAAATCGTGTATACCCCTATTTCTACACCAGATGGTACACTCGTCAAGAAATTCAAAGGAAACAATAGCGGACAGCCATCAACTGTTGTA

GACAACACGCTCATGGTCATACTGGCAGTCAACTATTCACTCAAGAAAAGTGGAATTCCAAGTGAGCTGCGCGACA

.((((.(...(((((((((((.(((((((((((.((((((.(((((((.(((((((.....((((..(((.((.((((.......))))....)))))..))))..((((((..(((((....((.

(((((.((.(((((((.((.....))........(((((((.(....))))))))))))))).))...(((....))).))))).))....)))))...(....)...)))))).)))))))).).))))))

).....)))))))...)))).))))))).).....((((((.......))))))))))))).)))))..).)))).

*GTTGGGATCGCTTGTTGGAGT**

****

****

rsa-miRn22

TTTTTTTTTTTTTTTTTTTCTTTTTTTTTTTTTTTTTTTTTTTTTTTAACTAAGATTAATCATTATCATTATTATTCAATGGTAGCCAGAGAAACACAAAGATCCAAACGAAGAAGAAGAACGGGAACAA

AGAAAAAAAACACAA

((((((((((.((((..((((((((((((.(((...((((((((((((......((....)).((((((((.......))))))))..))))))))...)))))...))).))))))))))))..)))).))

)))))))))......

**AGAAGAACGGGAACAA

AGAAA**********

rsa-miRn23

CTCCACGCGTATCCCGAAAGTCCAGCAGCTTCTTCAGGATTTCTTCAATGGGAAAGAGCTTTGCAAGTCTATTAACCCTGACGAGGCTGTGGCTTACGGTGCTGCGGTTCA

.....((((((..(((.(((.(((.(((((((.(((((.(((((......))))))..((...))............))))).))))))))))))).)))))).))).....

***********TCCCGAAAGTCCAGCAGCTTCTTCA***

ACGAGGCTGTGGCTTACGGTG********

rsa-miRn24

TTGCGTCTTTAGGATTGAGTCTAGAAGCATATATGTTGCATTCATGCATTGGGAGCCAACCTTGTAAAGAGCTCATGCTAAGTACTCAATTTGATACCCTTTGTAATCATGACAAGTAGTTTGAGCACTCTT

TTGAAAACTTGTAGACTTCGAGCCTTGAAACTCCTCTTGAAATTCATTGAATGTTGAAACTTCTCATCTTTGAAGCCAACTCCAATCTTATTTGAACTGAATGAACTTAATGCTTCTTGCTTATGGTCTTTT
GTGTAC

.((((.(...((((((((((..((((((((.((.(((.((((((..((..(((((..........((((((((((.((((.....(((...((((((.....)).)))))))....)))).)))))..))))
)......((((.......))))........)))))..))......((((...((((...((((........)))).))))..))))..........))))))))).)))))))))).)))))..)))))..
))))).

**********AGGATTGAGTCTAGAAGCATA***
****
******

rsa-miRn25

ACTGTGAGTTTCAGAACGATATAAAAGATCATGGATACAGTAAAGAGATTTAACTCAAATGAGGCTAGTATATCTATGGCCTTTATATCGTATTCGAAATTGACAAT

..(((.((((((..(((((((((((.(..((((((((...((((....)))).(((....))).......))))))))..))))))))))).)..)))))).)))..

************AGAACGATATAAAAGATCATGG***

ATGGCCTTTATATCGTATTCGA*******

rsa-miRn26

TGGCTGGCTAGAGATCTCTTCGATCTCGATCTCCTCTTCAGAATCAGCAAGGGAGGGTGATTTGTTGCCCGAGGAAGAGGGGAGGATGGGTGGGTTTCTCCAGCGC

..(((((..((((((((..((.((((...((((((((((....((.((((.(((......))).))))..)).)))))))))))))).)).)))))))))))))..

GAGGGGAGGATGGGTGGGTTTC******

rsa-miRn27

AAACAAGTCGGCGTCCCCGACATGGTCGTCTTCCTCAACAAGGAGGATCAGGTCGACGACGCCGAGCTGCTC

...((..((((((((..((((.(((((.((((........)))).))))).))))..))))))))..))...

**********GCGTCCCCGACATGGTCGTCT***

AGGATCAGGTCGACGACGCCG*****

rsa-miRn28

ATCTAGCCATTACGGCGACTCCAGGTAAAGCTTCTTGGACGAAGGATGATTGAGTTTTATGGGTTTCTGAAGTCTCTCTGAGGAAGATTTAGTAGAGTTGGCGTTTGTTAACG

...((((....(((.((((((..(.((((.((((((((((...(((.((((.(......).)))))))...)))).....)))))).)))).).)))))).)))..))))...

GAAGATTTAGTAGAGTTGGCG********

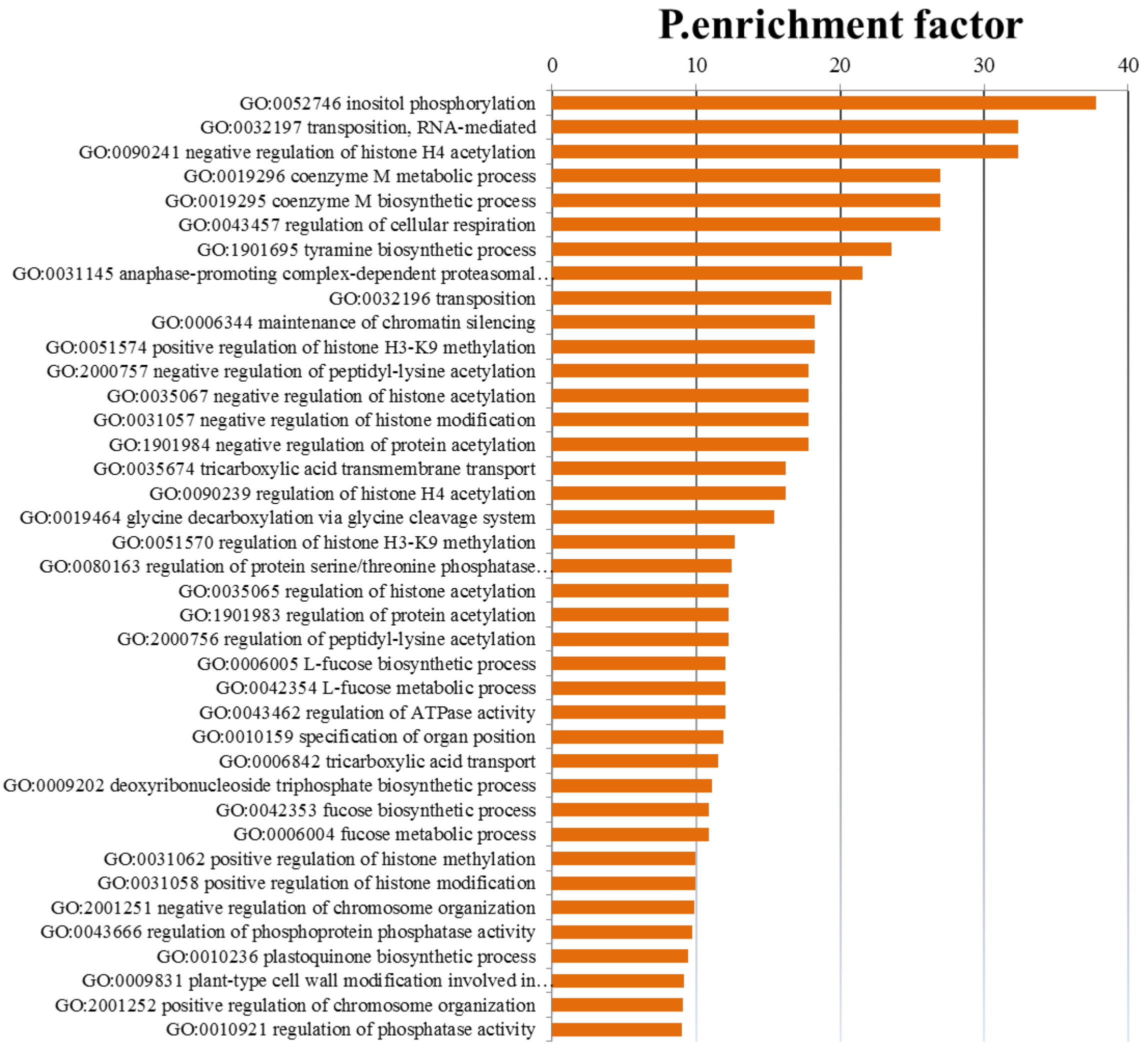

Supplementary Figure S3: GO enrichment multiple analysis in the classification of biological process.

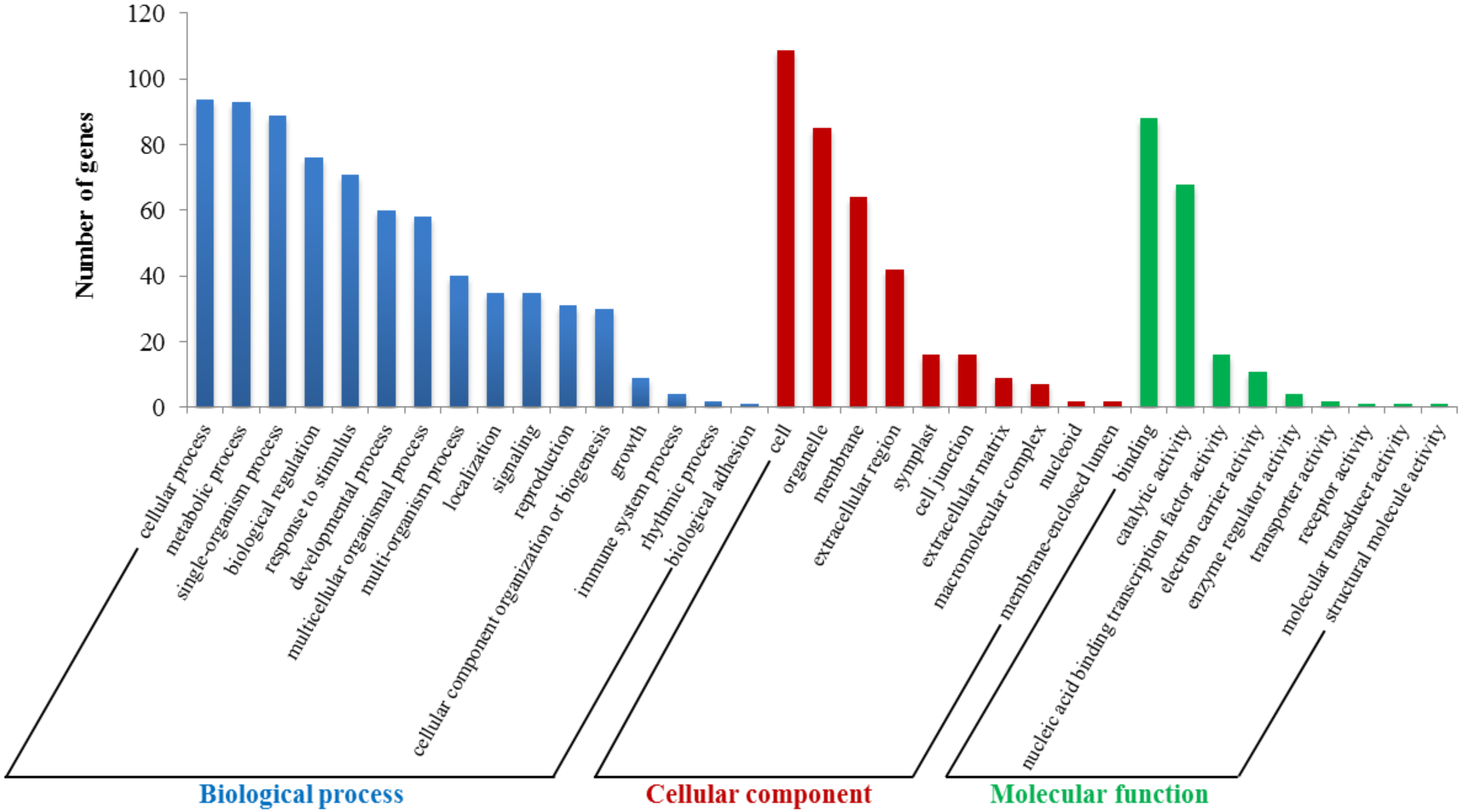

Supplementary Figure S4: GO classification of target genes for bolting and flowering-related miRNAs in radish.

2. Supplementary Tables

Supplementary Table S1: Primers of miRNAs and targets in radish for RT-qPCR.

Supplementary Table S2: Distribution of small RNAs among different categories in NAU-VS and NAU-RS libraries.

Supplementary Table S3: Detailed information of known miRNAs identified from NAU-VS and NAU-RS libraries.

Supplementary Table S4: Detailed information of novel miRNAs identified from NAU-VS and NAU-RS libraries.

Supplementary Table S5: Differentially expressed miRNAs between NAU-VS and NAU-RS in radish.

Supplementary Table S6: Summary of target genes from NAU-VS and NAU-RS libraries.

Supplementary Table S7: Putative targets of known and novel miRNAs identified in radish.

Supplementary Table S8: Predicted targets for unclassified non-conserved miRNAs in radish.

Supplementary Table S1: Primers of miRNAs and targets in radish for RT-qPCR.

miRNA / Target	Primer sequences (5'-3')
miR156a	TGACAGAAGAGAGTGAGCAC
miR159a	TTTGGATTGAAGGGAGCTCTA
miR172a	AGAATCTTGATGATGCTGCAT
miR164a	TGGAGAAGCAGGGCACGTGCA
miR167a	TGAAGCTGCCAGCATGATCTA
miR408-5p	ACAGGGAACAAGCAGAGCATG
miR535b	TGACGACCGAGAGAGCAAGAC
miR5227	TGAAGATGAAGAAGTTGAGCA
miR824	TAGACCATTTGTGAGAAGGGA
rsa-miRn3	CATTGACTGTATGCATTGGGAG
rsa-miRn9	ATGTGGGATGTGATTGTCAAG
rsa-miRn11	ATGCCTGGCTCCCTGTATGCC
SPL13-CL2234-F	CTTCTGTCATCATCTTCCT
SPL13-CL2234-R	CCTACTACTACTACTACTATTCC
SPL9-EX886942-F	CAGAGGTTATGGACACTAA
SPL9-EX886942-R	GGTTTGACAGAAGAGAGA
SPL6-Unigene3780-F	GAGTCTGGTGTATCTTATATTTG
SPL6-Unigene3780-R	TTGGTTGAGTGATTGAGAA
TOC1-FD977501-F	GCTTCCTATTCACATACCTA
TOC1-FD977501-R	TCCATCATCATCATCATCAT
MYB65-CL9825-F	ATTATTGGAACACTCGTATCA
MYB65-CL9825-R	ACAGGACATCATCATTAGC
AP2-FD572123-F	CAAGATGAACCAACAAGATT
AP2-FD572123-R	TGATGAGAAGTAGGAGTAGA
ARF8-Unigene18528-F	GAGAAGAGTTAGGGAAGATG
ARF8-Unigene18528-R	GCCAAGGAGAAGAATATCA
SPY-CL1667-F	AGCCAACGAATCAGTAAT
SPY-CL1667-R	CATACAGCGAACAACAAG
ARF16-FD576484-F	CACCTCAACCATCATCAA
ARF16-FD576484-R	CCCTTTCTCGTTTCTGTTA
ARF17-EX896877-F	TTACAACAACAACAACAACT

ARF17-EX896877-R	GGGAAGAGGACTAAAGAAG
VRN1-CL5009-F	TTAGATACGAAGGCAACTC
VRN1-CL5009-R	TACACCGAAGAAGAAGAAG
AGL16-Unigene1577-F	GGTTGGTGTCATCATCTT
AGL16-Unigene1577-R	TGGCATCTCTGTATCTCT
5.8S rRNA	ACGTCTGCCTGGGTGTCACAA

Supplementary Table S2: Distribution of small RNAs among different categories in NAU-VS and NAU-RS libraries.

Category	NAU-VS		NAU-RS	
	Unique	Total	Unique	Total
Total sRNAs	4,992,584	18,948,210	3,491,761	17,893,663
miRNA	61,574	2,174,0859	50,222	2,301,592
rRNA	67,035	1,695,1929	52,333	1,108,327
snRNA	5,347	21,182	5,453	20,696
snoRNA	2,914	8,044	1,268	3,017
tRNA	9,642	327,472	7,131	140,078
Unannotated	4,846,072	14,722,235	3,375,354	14,319,953

Supplementary Table S3: Detailed information of known miRNAs identified from NAU-VS and NAU-RS libraries.

Members	Sequences (5'-3')	Size	Arm	LP	MFE	NAU-VS		NAU-RS	
						Reads	Normalized	Reads	Normalized
**Conserved**									
miR156	CTGACAGAAGAGAGTGAGCAC	21	5p	122	-54.6	38	2.01	27	1.51
miR156a	TGACAGAAGAGAGTGAGCAC	20	5p	105	-45.9	78,246	4129.47	23,708	1324.94
miR156c	TTGACAGAAGAAAGAGAGCAC	21	5p	107	-40.7	34	1.79	40	2.24
miR156c-3p	GCTCACTGCTCTATCTGTCAGA	22	3p	102	-50	25	1.32	86	4.81
miR156d	TTGACAGAAGAGAGGGAGCAC	21	5p	103	-445.7	42	2.22	74	4.14
miR156g	ACAGAAGATAGAGAGCACAGA	21	5p	105	-50.7	59	3.11	101	5.64
miR156k	TTGACAGAAGAGAGTGAGCAC	21	5p	117	-51.2	51,438	2714.66	63,851	3568.36
miR156k-5p	TGACAGAAGAGAGCGAGCAC	20	5p	113	-46.1	78	4.12	54	3.02
miR157a	TTGACAGAAGATAGAGAGCAC	21	5p	98	-48.3	136,307	7193.66	62,008	3465.36
miR157a-3p	GCTCTCTAGCCTTCTGTCATC	21	3p	123	-54.9	296	15.62	410	22.91
miR157d	TGACAGAAGATAGAGAGCAC	20	5p	188	-61.8	283	14.94	184	10.28
miR158a	TCCCAAATGTAGACAAAGCA	20	3p	87	-33.8	137,925	7279.05	23,636	1320.91
miR158b	CCCCAAATGTAGACAAAGCA	20	3p	85	-30.7	26	1.37	15	0.84
miR158b-3p	TCCCAAATGTAGACAAAGC	19	3p	130	-41.26	138,085	7287.50	23,549	1316.05
miR159a	TTTGGATTGAAGGGAGCTCTA	21	3p	184	-75.9	1,710	90.25	1,489	83.21
miR159a-5p	GAGCTCCTTTAAGTTCAAACG	21	5p	219	-80.5	48	2.53	84	4.69
miR159b	CTTGCATATCTCAGGAGCTTT	21	3p	214	-93.81	218	11.51	133	7.43
miR159c-5p	AGCTCCCTTCGTCCAAAACG	20	5p	233	-98.3	22	1.16	40	2.24
miR160a	TGCCTGGCTCCCTGTATGCCA	21	5p	121	-48.10	441	23.27	172	9.61
miR160a-3p	GCGTATGAGGAGCCAAGCATA	21	3p	121	-50.80	3,149	166.19	16,791	938.38
miR160b	ATGCCTGGCTCCCTGTATGCC	21	5p	121	-48.10	181	9.55	474	26.49
miR160b-3p	GCGTACAGAGTAGTCAAGCATG	22	3p	122	-52.2	9,186	484.80	4,322	241.54
miR160d-3p	CGTACGAGGAGCCAAGCATGA	21	3p	106	-50.6	2,574	135.84	4,204	234.94
miR162a-5p	GGAGGCAGCGGTTCATCGATC	21	5p	141	-46.80	429	22.64	1,586	88.63

miR162a	TCGATAAACCTCTGCATCCAG	21	3p	141	-46.80	975	51.46	456	25.48
miR162-5p	TGGAGGCAGCGGTTCATCGATC	22	5p	141	-46.80	96	5.07	116	6.48
miR162-3p	GATCGATAAACCTCTGCATCC	21	3p	142	-47.26	468	24.70	505	28.22
miR164a	TGGAGAAGCAGGGCACGTGCA	21	5p	118	-45.00	25,312	1335.85	7,688	429.65
miR164b-3p	TGGAGAAGCAGGGCACGTGCG	21	3p	100	-46.2	426	22.48	1,220	68.18
miR165a-3p	TCGGACCAGGCTTCATCCCC	21	3p	145	-54.5	698	36.84	568	31.74
miR165a-5p	GAATGTTGTCTGGATCGAGGA	20	5p	151	-50.8	26	1.37	25	1.40
miR166	TCGGACCAGGCTTCATTCCCCT	21	3p	128	-44.6	409	21.59	376	21.01
miR166a	TCGGACCAGGCTTCATTCCCC	22	3p	171	-69.13	227,769	12020.61	201,536	11262.98
miR166b-5p	GGACTGTTGTCTGGCTCGAGG	21	5p	131	-44.9	4,546	239.92	8510	475.59
miR166d-5p	GGAATGTTGTCTGGCTCGAGG	21	5p	171	-69.13	38,182	2015.07	59,502	3325.31
miR166e	GGACCAGGCTTCATTCCCC	21	3p	171	-69.13	25	1.32	28	1.56
miR166g-5p	GGAATGTTGTTTGGCTCGAAG	21	5p	143	-53.5	850	44.86	1,536	85.84
miR166h-3p	TCGGACCAGGCTTCATTCCC	20	3p	171	-69.13	297	15.67	509	28.45
miR167	TCAAGCTGCCAGCATGATCTA	21	5p	68	-31.5	34	1.79	18	1.01
miR167a	TGAAGCTGCCAGCATGATCTA	21	5p	139	-56.90	398,555	21033.91	73,975	4134.15
miR167a-3p	GATCATGTTCGTAGTTTCACC	21	3p	137	-59.9	6,905	364.41	6,231	348.22
miR167b	TGAAGCTGACAGCATGATCTA	21	5p	90	-33.1	20	1.06	12	0.67
miR167d	TGAAGCTGCCAGCATGATCT	22	5p	323	-100.5	2,256	119.06	1,928	107.75
miR168	TCGCTTGGTGCAGGTCGGGA	20	5p	142	-73.60	2,750	145.13	2,575	143.91
miR168a	TCGCTTGGTGCAGGTCGGGAC	21	5p	142	-73.60	177,995	9393.76	156,597	8751.53
miR168a-3p	CCCGCCTTGCATCAACTGAAT	21	3p	142	-73.60	1,647	86.92	3,371	188.39
miR168b	TCGCTTGGTGCAGGTCGGG	19	5p	142	-73.60	13	0.69	23	1.29
miR169a	CAGCCAAGGATGACTTGCCGA	21	5p	142	-73.6	151	7.97	116	6.48
miR169d	TGAGCCAAGGATGATTTGCCG	21	5p	184	-61.1	57	3.01	29	1.62
miR169m	TGAGCCAAAGATGACTTGCCG	21	5p	98	-43.5	1,678	88.56	98	5.48
miR171b-5p	AGATATTAGTGCGGTTCAATC	21	5p	123	-42.7	2,940	155.16	1,276	71.31
miR172a	AGAATCTTGATGATGCTGCAT	21	3p	126	-58.9	73,506	3879.31	20,914	1168.79

miR172a-5p	GTGGCATCATCAAGATTCACA	21	5p	135	-58.9	37	1.95	22	1.23
miR172b-5p	GCAGCACCATTAAGATTCACA	20	5p	135	-54.8	7,329	386.79	5,302	296.31
miR172c	AGAATCTTGATGATGCTGCAG	21	3p	128	-50.7	12	0.63	8	0.45
miR172e	GGAATCTTGATGATGCTGCAT	21	3p	98	-47.9	28	1.48	15	0.84
miR390a	AAGCTCAGGAGGGATAGCGCC	21	5p	125	-58.6	6,584	347.47	2,210	123.51
miR390b-5p	TTAGCTCAGGAGGGATAGACA	21	5p	103	-39.2	49	2.59	26	1.45
miR390b-3p	CGCTATCCATCCTGAGTTCCA	21	3p	127	-51	181	9.55	21	1.17
miR391	TTCGCAGGAGAGATAGCGCCA	21	5p	96	-42.3	25,425	1341.82	8,072	451.11
miR391-3p	ACGGTATCTCTCCTACGCAAC	21	3p	103	-41.8	350	18.47	1,153	64.44
miR393a	TCCAAAGGGATCGCATTGATCC	21	5p	160	-53.4	22	1.16	13	0.73
miR395b	CTGAAGTGTTTGGGGGGACTC	21	3p	115	-54.9	681	35.94	160	8.94
miR396a	TTCCACAGCTTTCTTGAACTG	21	5p	153	-57.6	637	33.62	148	8.27
miR396b	TTCCACAGCTTTCTTGAACTT	21	5p	147	-39.04	189	9.97	420	23.47
miR396b-3p	GCTCAAGAAAGCTGTGGGAAA	21	3p	147	-39.04	4,260	224.82	5,986	334.53
miR397a	TCATTGAGTGCAGCGTTGATGT	22	5p	108	-40	854	45.07	633	35.38
miR398a-5p	GGAGTGTCATGAGAACACAGA	21	5p	102	-37	29	1.53	47	2.63
miR398c-5p	GGGTCGATATGAGAACACATG	21	5p	114	-48.10	382	20.16	30,020	1677.69
miR399b	TGCCAAAGGAGAGTTGCCCTG	21	3p	135	-61	154	8.13	130	7.27
miR399d	TGCCAAAGGAGATTTGCCCCG	22	3p	105	-46.4	15	0.79	28	1.56
miR399d-5p	GGGCGGATACTCCTGTGGCAGA	22	5p	129	-60.3	175	9.24	572	31.97
miR399g	GGGCTTCTCTCTATTGGCAGG	21	5p	224	-65	35	1.85	26	1.45
miR399h-5p	GGGCAAGATCTCTATTGGCAGG	22	5p	138	-59.1	213	11.24	373	20.85
miR408-5p	ACAGGGAACAAGCAGAGCATG	21	5p	110	-46.10	4,544	239.81	740,985	41410.47
**Non-Conserved**									
miR1885a	TCAATGAAAGGTATGATTCCCT	22	3p	196	-73.6	18	0.95	30	1.68
miR1885b	TACATCTTCTCCGCGGAAGCTC	22	3p	325	-129.1	8,319	439.04	12,339	689.57
miR1863	TGGCTCTGATACCATGTAGAT	21	3p	143	-35.5	9,168	483.85	2,416	135.02
miR2111a-3p	GACCTCAGGATGCGGATTACC	21	3p	159	-67.3	31	1.64	179	10.00

miR2111c	TAATCTGCATCCTGGGGTTTA	21	5p	153	-83.3	82	4.33	459	25.65
miR394a	TTGGCATTCTGTCCACCTCC	20	5p	138	-54.6	20	1.06	8	0.45
miR394a-3p	AGGTGGGCATACTGCCAATAG	21	3p	126	-50.61	48	2.53	11	0.61
miR394b-3p	AGGTGGTCATACTGTCAACA	20	5p	142	-39.50	4	0.21	36	2.01
miR400	TATGAGAGTATTATAAGTCAC	21	5p	122	-47.5	91	4.80	94	5.25
miR400-3p	GACTTATAATGATCTCATGAA	21	3p	122	-47.5	39	2.06	147	8.22
miR403	TTAGATTCACGCACAAACTCG	21	3p	102	-36.3	1,900	100.27	1,485	82.99
miR535b	TGACGACCGAGAGAGCAAGAC	21	5p	95	-61.4	0	0.01	2,797	156.31
miR5161	TCTGGATAGAAGGTAGGATA	20	3p	276	-62.4	1,423	75.10	5,868	327.94
miR5227	TGAAGATGAAGAAGTTGAGCA	21	5p	137	-26	144	7.60	0	0.01
miR6273	AAAGGGCTGATTTTTTTTT	19	3p	123	-42.2	0	0.01	360	20.12
miR6284	TATGACCAGGATGAAGCTT	19	3p	97	-31.9	163	8.60	0	0.01
miR824	TAGACCATTTGTGAGAAGGGA	21	5p	689	-198.82	1,557	82.17	521	29.12
miR824-3p	CCTTCTCATTCGATGGTCTAGA	22	3p	77	-22.80	265	13.99	185	10.34
miR860	TCAATACATTGGACTACATAT	21	3p	108	-58.20	212	11.19	653	36.49

LP (nt): The length of precursor, *MFE (kcal/mol):* minimal folding free energy.

Supplementary Table S4: Detailed information of novel miRNAs identified from NAU-VS and NAU-RS libraries.

miRNAs	Sequences (5'-3')	Size	Arm	LP	MFE	NAU-VS		NAU-RS		Location
						Reads	Normalized	Reads	Normalized	
rsa-miRn1	AGAAGAGGAAGAGGATGAAGAT	22	5p	209	-75.8	0	0.01	525	29.34	EY947932
rsa-miRn2a	AGGGGAGGATGGGTGGGTTTC	21	3p	106	-44.2	0	0.01	168	9.39	FD986211
rsa-miRn2b	AGGGGAGGATGGGTGGGTTTC	21	3p	111	-45	0	0.01	168	9.39	FD958546
rsa-miRn3	CATTGACTGTATGCATTGGGAG	22	3p	272	-73.2	919	48.5	86	4.81	CL1672.Contig1
rsa-miRn3*	ACAAAAGTGTACTGCGGCAATTCG	22	5p	272	-73.2	1	0.05	0	0	CL1672.Contig1
rsa-miRn4	TCGGAATTCCGTCGGAATATA	21	3p	100	-56.31	743	39.21	1,402	78.35	CL2831.Contig4
rsa-miRn4*	TATTCCGACGACAATTCCGACG	21	5p	100	-56.31	4	0.21	21	1.17	CL2831.Contig4
rsa-miRn5	TACCGATAGATGTGGAAGCGT	21	3p	184	-75.8	2,502	132.04	4,596	256.85	CL2916.Contig2
rsa-miRn5*	GCTTCCATATCTAGCAGTAGG	21	5p	184	-75.8	7	0.37	34	1.9	CL2916.Contig2
rsa-miRn6	TTTGCGTGAGTATGTGGATGT	21	5p	119	-49	2,809	148.25	1,341	74.94	CL4600.Contig2
rsa-miRn6*	ATCCACATACTCACGAAAATC	21	3p	119	-49	10	0.53	25	1.4	CL4600.Contig2
rsa-miRn7a	AGCAAACGAGAATTGAACGGA	21	3p	106	-47.24	842	44.44	1,018	56.89	FD935332
rsa-miRn7a*	CGTTCAGTTCTCCTTTTGCTTC	21	5p	106	-47.24	1	0.05	3	0.17	FD935332
rsa-miRn7b	AGCAAACGAGAATTGAACGGA	21	3p	114	-51.6	842	44.44	1,018	56.89	CL5135.Contig1
rsa-miRn7b*	CGTTCAGTTCTCCTTTTGCTTC	21	5p	114	-51.6	1	0.05	3	0.17	CL5135.Contig1
rsa-miRn8	CAGAACGATATAAAAGATCATGG	23	5p	109	-30.2	0	0.01	40	2.24	CL6156.Contig1
rsa-miRn8*	ATGGCCTTTATATCGTATTCGAA	23	3p	109	-30.2	0	0.01	8	0.45	CL6156.Contig1
rsa-miRn9	ATGTGGGATGTGATTGTCAAG	21	3p	83	-22.36	112	5.91	319	17.83	Unigene19102
rsa-miRn10	GTGACCGGCGCGTGGCGGCTC	21	5p	74	-36.9	15	0.79	9	0.5	Unigene19802
rsa-miRn11	ATGCCTGGCTCCCTGTATGCC	21	5p	106	-49.3	0	0.01	457	25.54	EY928785
rsa-miRn12	GGTAGTTTGACCGCGAAATTT	21	3p	143	-28	6,902	364.26	11,143	622.73	EW722117
rsa-miRn13	GTCTGTATGGTATGGGTGGAGG	22	5p	110	-52.11	0	0.01	19	1.06	Unigene29247
rsa-miRn14	GCTAATGAGATCGAAATACTGA	22	5p	182	-28.9	0	0.01	23	1.29	Unigene7309
rsa-miRn14*	AGGTTTCGGTTCGGTTAGTTG	22	3p	182	-28.9	0	0.01	1	0.06	Unigene7309

rsa-miRn15a	CAGAGACAGAGAGGAGAAAGGAA	23	5p	90	-34	22	1.16	28	1.56	Unigene205
rsa-miRn15b	CAGAGACAGAGAGGAGAAAGGAA	23	5p	94	-34.2	22	1.16	28	1.56	FD957237
rsa-miRn16	ATATACTGAAGTTTATACTCT	21	5p	208	-37	0	0.01	18	1.01	EY928450
rsa-miRn17	TATGAATGATGCGGGAGATGT	21	3p	112	-33.5	108	5.7	17	0.95	FY434434
rsa-miRn17*	ATCTGCCGACTCATCCATCCA	21	5p	112	-33.5	3	0.16	3	0.17	FY434434
rsa-miRn18	AGGACCAGGTCGACGACGCCG	21	3p	80	-44.9	312	16.47	558	31.18	FY437994
rsa-miRn18*	GCGTCCCCGACATGGTCGTCT	21	5p	80	-44.9	17	0.9	81	4.53	FY437994
rsa-miRn19	TGAGCGGCAACTATTGTAGGT	21	5p	111	-50.7	14	0.74	9	0.5	FY434072
rsa-miRn20	TCTTGAGTTCGAGGGACGCCA	21	3p	107	-65.6	90	4.75	153	8.55	FY453420
rsa-miRn20*	ACGTTTCTCGAACTCAAGACC	21	5p	107	-65.6	2	0.11	1	0.06	FY453420
rsa-miRn21	GTTGGGATCGCTTGTTGGAGT	21	5p	340	-104.4	798	42.11	0	0.01	Unigene13277
rsa-miRn22	AGAAGAACGGGAACAAAGAAA	22	3p	147	-23.6	0	0.01	22	1.23	EY947898
rsa-miRn23	ACGAGGCTGTGGCTTACGGTG	21	3p	111	-37.1	0	0.01	26	1.45	FD556890
rsa-miRn23*	TCCCGAAAGTCCAGCAGCTTCTTCA	21	5p	111	-37.1	0	0.01	1	0.06	FD556890
rsa-miRn24	AGGATTGAGTCTAGAAGCATA	21	5p	270	-57.1	127	6.7	0	0.01	FD936100
rsa-miRn25	AGAACGATATAAAAGATCATGG	22	3p	107	-30.2	48	2.53	0	0.01	CL6156.Contig1
rsa-miRn25*	ATGGCCTTTATATCGTATTCGA	22	3p	107	-30.2	20	1.06	0	0.01	CL6156.Contig1
rsa-miRn26a	GAGGGGAGGATGGGTGGGTTTC	22	3p	106	-44.2	82	4.33	0	0.01	FD986211
rsa-miRn26b	GAGGGGAGGATGGGTGGGTTTC	22	3p	111	-45	82	4.33	0	0.01	FD958546
rsa-miRn27	GCGTCCCCGACATGGTCGTCT	21	5p	72	-31.7	0	0.01	81	4.53	CL7005.Contig2
rsa-miRn27*	AGGATCAGGTCGACGACGCCG	21	3p	72	-31.7	0	0.01	5	0.28	CL7005.Contig2
rsa-miRn28	GAAGATTTAGTAGAGTTGGCG	21	3p	113	-27.3	0	0.01	37	2.07	FY451021

LP (nt): The length of precursor, *MFE (kcal/mol):* minimal folding free energy.

Supplementary Table S5: Differentially expressed miRNAs between NAU-VS and NAU-RS in radish.

Family	miRNA name	NAU-VS		NAU-RS		Fold change log_2(NAU-VS/ NAU-RS)	Regulate mode	*P*-value	Sig-lable
		Reads	Normalized	Reads	Normalized				
**Conserved**									
miR156	miR156a	78,246	4129.47	23,708	1324.94	-1.64	down	0	**
	miR156c-3p	25	1.32	86	4.81	1.87	up	5.11E-10	**
	miR157a	136,307	7193.66	62,008	3465.36	-1.05	down	0	**
miR158	miR158a	137,925	7279.05	23,636	1320.91	-2.46	down	0	**
	miR158b-3p	138,085	7287.50	23,549	1316.05	-2.47	down	0	**
miR160	miR160a	441	23.27	172	9.61	-1.28	down	4.44E-25	**
	miR160a-3p	3,149	166.19	16,791	938.38	2.50	up	0	**
	miR160b	181	9.55	474	26.49	1.47	up	4.04E-35	**
	miR160b-3p	9,186	484.80	4,322	241.54	-1.01	down	0	**
miR162	miR162a	975	51.46	456	25.48	-1.01	down	1.61E-37	**
	miR162a-5p	429	22.64	1,586	88.63	1.97	up	2.21E-170	**
miR164	miR164a	25,312	1335.85	7,688	429.65	-1.64	down	0	**
	miR164b-3p	426	22.48	1,220	68.18	1.60	up	5.45E-99	**
miR167	miR167a	398,555	21033.91	73,975	4134.15	-2.35	down	0	**
miR168	miR168a-3p	1,647	86.92	3,371	188.39	1.12	up	7.99E-156	**
miR169	miR169m	1,678	88.56	98	5.48	-4.02	down	0	**
miR171	miR171b-5p	2,940	155.16	1,276	71.31	-1.12	down	8.59E-129	**
miR172	miR172a	73,506	3879.31	20,914	1168.79	-1.73	down	0	**
miR390	miR390a	6,584	347.47	2,210	123.51	-1.49	down	0	**
	miR390b-3p	181	9.55	21	1.17	-3.02	down	3.14E-31	**
miR391	miR391	25,425	1341.82	8,072	451.11	-1.57	down	0	**
	miR391-3p	350	18.47	1,153	64.44	1.80	up	1.55E-110	**

miR395	miR395b	681	35.94	160	8.94	-2.01	down	4.52E-71	**
miR396	miR396a	637	33.62	148	8.27	-2.02	down	2.85E-67	**
	miR396b	189	9.97	420	23.47	1.23	up	2.73E-24	**
miR398	miR398c-5p	382	20.16	30,020	1677.69	6.38	up	0	**
miR399	miR399d-5p	175	9.24	572	31.97	1.79	up	2.86E-55	**
miR408	miR408-5p	4,544	239.81	740,985	41410.47	7.43	up	0	**
**Non-conserved**									
miR1863	miR1863	9,168	483.85	2,416	135.02	-1.84	down	0	**
miR2111	miR2111a-3p	31	1.64	179	10.00	2.61	up	1.26E-28	**
	miR2111c	82	4.33	459	25.65	2.57	up	1.35E-69	**
miR394	miR394a	20	1.06	8	0.45	-1.24	down	0.035540601	*
	miR394a-3p	48	2.53	11	0.61	-2.04	down	2.22E-06	**
	miR394b-3p	4	0.21	36	2.01	3.25	up	3.90E-08	**
miR400	miR400-3p	39	2.06	147	8.22	2.00	up	1.52E-17	**
miR535	miR535b	0	0.01	2,797	156.31	13.93	up	0	**
miR5161	miR5161	1,423	75.10	5,868	327.94	2.13	up	0	**
miR5227	miR5227	144	7.60	0	0.01	-9.57	down	2.68E-42	**
miR6273	miR6273	0	0.01	360	20.12	10.97	up	1.19E-113	**
miR6284	miR6284	163	8.60	0	0.01	-9.75	down	8.75E-48	**
miR824	miR824	1,557	82.17	521	29.12	-1.50	down	6.39E-107	**
miR860	miR860	212	11.19	653	36.49	1.71	up	8.61E-59	**
**Novel**									
rsa-miRn1	rsa-miRn1	0	0.01	525	29.34	11.52	up	2.11E-165	**
rsa-miRn2	rsa-miRn2a	0	0.01	168	9.39	9.87	up	1.97E-53	**
rsa-miRn3	rsa-miRn3	919	48.5	86	4.81	-3.34	down	8.24E-167	**
rsa-miRn8	rsa-miRn8	0	0.01	40	2.24	7.8	up	2.76E-13	**
rsa-miRn9	rsa-miRn9	112	5.91	319	17.83	1.59	up	5.95E-27	**
rsa-miRn11	rsa-miRn11	0	0.01	457	25.54	11.32	up	4.5E-144	**

rsa-miRn13	rsa-miRn13	0	0.01	19	1.06	6.73	up	1.07E-06	**
rsa-miRn14	rsa-miRn14	0	0.01	23	1.29	7.01	up	5.94E-08	**
rsa-miRn16	rsa-miRn16	0	0.01	18	1.01	6.65	up	2.20E-06	**
rsa-miRn17	rsa-miRn17	108	5.7	17	0.95	-2.58	down	1.71E-16	**
rsa-miRn22	rsa-miRn22	0	0.01	22	1.23	6.94	up	1.22E-07	**
rsa-miRn23	rsa-miRn23	0	0.01	26	1.45	7.18	up	6.8E-09	**
rsa-miRn24	rsa-miRn24	127	6.7	0	0.01	-9.39	down	2.18E-37	**
rsa-miRn25	rsa-miRn25	48	2.53	0	0.01	-7.98	down	1.42E-14	**
rsa-miRn26	rsa-miRn26a	82	4.33	0	0.01	-8.76	down	2.15E-24	**
rsa-miRn27	rsa-miRn27	0	0.01	81	4.53	8.82	up	3.82E-26	**
rsa-miRn28	rsa-miRn28	0	0.01	37	2.07	7.69	up	2.41E-12	**

Supplementary Table S6: Summary of target genes from NAU-VS and NAU-RS libraries.

Sample	miRNA number			Target gene number			Target location number		
	Known	Novel	Total	Known	Novel	Total	Known	Novel	Total
NAU-VS	121	40	161	1503	277	1780	1659	334	1993
NAU-RS	111	52	163	2111	500	2611	2369	651	3020
NAU-RS & NAU-VS	110	14	124	1025	31	1056	1148	36	1184
Two libraries total	122	78	200	2589	746	3335	2880	949	3829
Differences between two libraries	11	64	75	1564	715	2279	1732	913	2645

Supplementary Table S7: Putative targets of known and novel miRNAs identified in radish.

Family	miRNA	Target sequence	Gene ID	Target gene	Target gene annotation
**Conserved**					
miR156/157	miR156a	CL289.Contig1	AT3G15270	*SPL5*	Squamosa promoter-binding-like protein 5
		CL2234.Contig1	AT5G50670	*SPL13*	Squamosa promoter-binding-like protein 13
		Unigene3780	AT1G69170	*SPL6*	Squamosa promoter-binding-like protein 6
		Unigene8578	AT3G56100	*MRLK*	Meristematic receptor-like kinase
		Unigene9933	AT3G57920	*SPL15*	Squamosa promoter-binding-like protein 15
		Rsa#S41982434	AT3G20080	*CYP705A15*	Cytochrome P450, family 705, subfamily A, polypeptide 15
		Rsa#S43017568	AT2G33810	*SPL3*	Squamosa promoter-binding-like protein 3
		Rsa#S41982434	AT2G33810	*SPL3*	Squamosa promoter-binding-like protein 3
		FD977501	AT5G61380	*TOC1*	Two-component response regulator-like APRR1
		EX886942	AT2G42200	*SPL9*	Squamosa promoter-binding-like protein 9
	miR156k	CL289.Contig1	AT3G15270	*SPL5*	Squamosa promoter-binding-like protein 5
		CL2234.Contig1	AT5G50670	*SPL13*	Squamosa promoter-binding-like protein 13
		CL2234.Contig2	AT5G50670	*SPL13*	Squamosa promoter-binding-like protein 13
		CL2234.Contig3	AT5G50670	*SPL13*	Squamosa promoter-binding-like protein 13
		Unigene3780	AT1G69170	*SPL6*	Squamosa promoter-binding-like protein 6
		Unigene9933	AT3G57920	*SPL15*	Squamosa promoter-binding-like protein 15
		Rsa#S43017568	AT2G33810	*SPL3*	Squamosa promoter-binding-like protein 3
	miR157a	CL2234.Contig1	AT5G50670	*SPL13*	Squamosa promoter-binding-like protein 13
		CL2234.Contig2	AT5G50670	*SPL13*	Squamosa promoter-binding-like protein 13
		CL2234.Contig3	AT5G50670	*SPL13*	Squamosa promoter-binding-like protein 13
		Unigene3780	AT1G69170	*SPL6*	Squamosa promoter-binding-like protein 6
		Unigene9933	AT3G57920	*SPL15*	Squamosa promoter-binding-like protein 15

miR158	miR158a	Rsa#S42049270	AT1G64100		Pentatricopeptide repeat-containing protein
	miR158b-3p	Rsa#S42049270	AT1G64100		Pentatricopeptide repeat-containing protein
miR159	miR159a	CL3756.Contig1	AT1G77860	*KOM*	Protein KOMPEITO
		CL2461.Contig1	AT1G53190		RING/U-box domain-containing protein
		CL2461.Contig3	AT1G03190	*UVH6*	DNA repair helicase UVH6
		CL8717.Contig1	AT4G27330	*SPL*	Putative transcription factor SPL
		CL9407.Contig3	AT5G10200		ARM-repeat/tetratricopeptide repeat-like protein
		CL9825.Contig1	AT3G11440	*MYB65*	MYB domain protein 65
		CL9825.Contig2	AT3G11440	*MYB65*	MYB domain protein 65
		Rsa#S42037487	AT2G32460	*MYB101*	MYB domain protein 101
		Rsa#S42034459	AT4G36410	*UBC17*	Putative ubiquitin-conjugating enzyme E2 17
		Rsa#S42591074	AT4G36410	*UBC17*	Putative ubiquitin-conjugating enzyme E2 17
		Rsa#S41979156	AT4G27330	*SPL*	Putative transcription factor SPL
		EY943953	AT5G10480	*PAS2*	(3R)-3-hydroxyacyl-[acyl-carrier protein] dehydratase PASTICCINO 2
miR160	miR160a	Unigene466	AT3G18440	*ALMT9*	Aluminum-activated malate transporter 9
		Rsa#S42581764	AT4G30080	*ARF16*	Auxin response factor 16
	miR160a-3p	EW732793	AT1G48020	*PMEI1*	Pectin methylesterase inhibitor AtPMEI1
		FD949106	AT1G48020	*PMEI1*	Pectin methylesterase inhibitor AtPMEI1
		EY930710	AT1G48020	*PMEI1*	Pectin methylesterase inhibitor AtPMEI1
		FD962300	AT1G48020	*PMEI1*	Pectin methylesterase inhibitor AtPMEI1
	miR160b	Rsa#S42581764	AT4G30080	*ARF16*	Auxin response factor 16
miR164	miR164a	Unigene2996	AT1G56010	*NAC1*	Transcription factor NAC1
		FY442454	AT3G12977		No apical meristem-domain containing transcriptional regulator
		EW715661	AT5G61430	*NAC100*	NAC domain containing protein 100
	miR164b-3p	Rsa#S43010415	AT1G72360	*HRE1*	Ethylene-responsive transcription factor ERF073
miR165/166	miR165a-3p	CL7974.Contig1	AT1G52150	*ATHB-15*	Homeobox-leucine zipper protein ATHB-15

miR167	miR167a	CL1667.Contig1	AT3G11540	*SPY*	putative UDP-N-acetylglucosamine-peptide N-acetylglucosaminyltransferase SPINDLY
		Unigene18528	AT5G37020	*ARF8*	Auxin response factor 8
		Unigene18529	AT5G37020	*ARF8*	Auxin response factor 8
miR169	miR169a	CL1568.Contig1	AT5G26751	*SK 11*	Shaggy-related protein kinase alpha
		CL1568.Contig2	AT5G26751	*SK 11*	Shaggy-related protein kinase alpha
	miR169m	FY429716	AT3G57520	*SIP2*	Putative galactinol-surose galactosyltransferase
		EX895539	AT1G61900		Hypothetical protein
miR171	miR171b-3p	CL5811.Contig1	AT4G00150	*HAM3*	Protein LOST MERISTEMS 3
miR172	miR172a	CL2600.Contig1	AT2G28550	*RAP2.7*	TARGET OF EARLY ACTIVATION TAGGED 1
		CL2600.Contig2	AT2G28550	*RAP2.7*	TARGET OF EARLY ACTIVATION TAGGED 1
		CL8288.Contig2	AT1G70290	*TPS8*	Putative alpha,alpha-trehalose-phosphate synthase [UDP-forming] 8
		CL8288.Contig3	AT1G70290	*TPS8*	Putative alpha,alpha-trehalose-phosphate synthase [UDP-forming] 8
		Rsa#S41997500	AT1G55310	*SR33*	SC35-like splicing factor 33
		Unigene11673	AT5G42950		GYF domain-containing protein
		Rsa#S42044145	AT2G42320		Nucleolar protein gar2-like protein
		EW732550	AT5G60120	*TOE2*	AP2-like ethylene-responsive transcription factor TOE2
		FD572123	AT4G36920	*AP2*	Floral homeotic protein APETALA 2
		EW719579	AT3G13570	*SCL30A*	SC35-like splicing factor 30A
miR391	miR391	CL175.Contig3	AT4G29900	*ACA10*	Calcium-transporting ATPase 10
		CL175.Contig4	AT4G29900	*ACA10*	Calcium-transporting ATPase 10
miR393	miR393a	Unigene359	AT1G12820	*AFB3*	Auxin signaling F-box 3 protein
		FD955493	AT3G62980	*TIR1*	Protein TRANSPORT INHIBITOR RESPONSE 1
		Rsa#S43016969	AT4G03190	*GRH1*	GRR1-like protein 1
miR395	miR395b	FY441630	AT5G43780	*APS4*	Sulfate adenylyltransferase
miR396	miR396b-3p	Unigene4815	AT5G15920	*SMC5*	Structural maintenance of chromosomes 5

		Rsa#S41989522	AT1G20540		Transducin/WD-40 repeat-containing protein
	miR396a	CL879.Contig1	AT2G40360		Transducin/WD-40 repeat-containing protein
		CL3298.Contig4	AT4G35240		Uncharacterized protein
		CL6202.Contig1	AT5G61440	*ACHT5*	Atypical CYS HIS rich thioredoxin 5
		CL6202.Contig2	AT5G61440	*ACHT5*	Atypical CYS HIS rich thioredoxin 5
	miR396b	CL6202.Contig1	AT5G61440	*ACHT5*	Atypical CYS HIS rich thioredoxin 5
		CL6202.Contig2	AT5G61440	*ACHT5*	Atypical CYS HIS rich thioredoxin 5
		Unigene20881	AT4G24610		Uncharacterized protein
		Unigene22800	AT3G01660		S-adenosylmethionine-dependent methyltransferase domain-containing protein
		Rsa#S42003511	AT3G01660		S-adenosylmethionine-dependent methyltransferase domain-containing protein
miR397	miR397a	CL379.Contig2	AT3G45010	*scpl48*	Serine carboxypeptidase-like 48
miR399	miR399d-5p	Unigene10472	AT2G46440	*CNGC11*	Cyclic nucleotide-gated channel 11
miR408	miR408-5p	CL13722.Contig2	AT4G28485	*DMP7*	DUF79 domain membrane protein 7
		CL13722.Contig5	AT2G20560		DNAJ heat shock family protein
**Non-conserved**					
miR1885	miR1885b	CL9579.Contig1	AT4G16890	*SNC1*	Protein SUPPRESSOR OF npr1-1, CONSTITUTIVE 1
		CL9922.Contig3	AT1G65850		TIR-NBS-LRR class disease resistance protein
		Unigene1615	AT1G72860		TIR-NBS-LRR class disease resistance protein
miR1863	miR1863	CL3633.Contig1	AT2G25660	*emb2410*	Embryo defective 2410 protein
		Unigene16275	AT1G21280		Hypothetical protein
miR394	miR394a	Unigene5626	AT1G27340		Protein LEAF CURLING RESPONSIVENESS
miR400	miR400	CL2666.Contig2	AT3G22470		Pentatricopeptide repeat-containing protein
miR403	miR403	CL3585.Contig3	AT1G31280	*AGO2*	Argonaute 2
		Rsa#S41987411	AT1G31280	*AGO2*	Argonaute 2
miR5161	miR5161	CL4253.Contig1	AT5G54250	*CNGC4*	Cyclic nucleotide-gated ion channel 4
		CL4854.Contig1	AT5G52870		Membrane-associated kinase regulator family protein

miR5227	miR5227	CL1189.Contig1	AT5G60550	*GRIK2*	Geminivirus rep interacting kinase 2
		CL1534.Contig12	AT2G29550	*TUB7*	Tubulin beta-7 chain
		CL2898.Contig1	AT3G10985	*SAG20*	Senescence associated protein 20
		CL5009.Contig1	AT3G18990	*VRN1*	B3 domain-containing transcription factor VRN1
		Rsa#S41998433	AT3G18990	*VRN1*	B3 domain-containing transcription factor VRN1
		CL7664.Contig1	AT3G11200	*AL2*	alfin-like 2 protein
		CL7684.Contig1	AT3G11330	*PIRL9*	Ras group-related LRR 9 protein
		CL9378.Contig1	AT1G09340	*CRB*	Chloroplast stem-loop binding protein
		Unigene19359	AT3G56150	*EIF3C*	Eukaryotic translation initiation factor 3 subunit C
		Rsa#S42008589	AT3G16950	*LPD1*	Lipoamide dehydrogenase 1
		Rsa#S43023896	AT3G45980	*HTB9*	Histone HTB9
		FD952118	AT5G22880	*HTB2*	Histone H2B
		Rsa#S42578477	AT4G20280	*TAF11*	TBP-associated factor 11
		FD951112	AT3G13320	*CAX2*	Low affinity calcium antiporter CAX2
miR6273	miR6273	CL2517.Contig2	AT1G04080	*PRP39*	Pre-mRNA-processing factor 39
		CL2918.Contig1	AT4G30890	*UBP24*	Ubiquitin carboxyl-terminal hydrolase 24
		Unigene20712	AT1G58200	*MSL3*	MSCS-like 3
		Unigene22978	AT1G74420	*FUT3*	Fucosyltransferase 3
		Unigene23968	AT2G34357		NUC173 domain-containing protein
		Unigene9692	AT1G55840		Sec14p-like phosphatidylinositol transfer family protein
		Unigene9776	AT3G61480		beta-chain like quinoprotein amine dehydrogenase
		Unigene13914	AT4G32020		Hypothetical protein
		Unigene25162	AT5G41130		Esterase/lipase/thioesterase family protein
miR6284	miR6284	CL5701.Contig1	AT5G17440		LUC7 related protein
		Unigene4264	AT2G07050	*CAS1*	Cycloartenol synthase
		Unigene17570	AT4G26640	*WRKY20*	Putative WRKY transcription factor 20

		Rsa#S42584834	AT1G55350	*DEK1*	Calpain-type cysteine protease DEK1
miR824	miR824	Unigene1577	AT3G57230	*AGL16*	Agamous-like MADS-box protein AGL16
		Rsa#S43011727	AT3G14560		Hypothetical protein
miR860	miR860	CL754.Contig1	AT4G14540	*NF-YB3*	Nuclear transcription factor Y subunit B-3
		CL2870.Contig2	AT5G26030	*FC1*	Ferrochelatase 1
**Novel**					
rsa-miRn2	rsa-miRn2a	EY934987	AT5G56890		Protein kinase family protein
		FY453870	AT3G26650	*GAPA*	Glyceraldehyde-3-phosphate dehydrogenase A subunit
rsa-miRn3	rsa-miRn3	EX907726	AT5G63180		Putative pectate lyase 22
		EW725360	AT2G13770		Hypothetical protein
		FY434255	AT4G24780		Putative pectate lyase 18
rsa-miRn9	rsa-miRn9	CL7101.Contig3	AT1G27900		RNA helicase family protein
rsa-miRn11	rsa-miRn11	FD576484	AT4G30080	*ARF16*	Auxin response factor 16
		EX896877	AT1G77850	*ARF17*	Auxin response factor 17
rsa-miRn14	rsa-miRn14	FY429806	AT5G58640		Selenoprotein, Rdx type
rsa-miRn16	rsa-miRn16	FD573405	AT3G08610		NADH dehydrogenase [ubiquinone] 1 alpha subcomplex 1
rsa-miRn17	rsa-miRn17	FD985437	AT4G26160	*ACHT1*	Atypical CYS HIS rich thioredoxin 1
rsa-miRn22	rsa-miRn22	CL151.Contig1	AT2G33120	*SAR1*	Vesicle-associated membrane protein 722
		Unigene6221	AT4G34870	*ROC5*	Rotamase cyclophilin 5
		Unigene20220	ArthMp017	*ccb452*	Cytochrome c biogenesis orf452
		Unigene29948	AT5G44550		Hypothetical protein
		FD578327	AT3G57630		Exostosin family protein
		EY947898	AT2G16600	*ROC3*	Cyclophilin ROC3
rsa-miRn23	rsa-miRn23	CL908.Contig13	AT2G37770		aldo-keto reductase family 4 member C9
		FD953729	AT5G05500		Pollen_Ole_e_I domain-containing protein
rsa-miRn26	rsa-miRn26	FD981451	AT2G33800		30S ribosomal protein S5

rsa-miRn27	rsa-miRn27	CL6997.Contig1	AT5G58520		Protein kinase family protein
		CL7005.Contig1	AT4G20360	*RABE1b*	Putative elongation factor Tu
		Unigene8608	AT1G20300		Pentatricopeptide repeat-containing protein
rsa-miRn28	rsa-miRn28	EX902723	AT1G06780	*GAUT6*	Probable galacturonosyltransferase 6

Supplementary Table S8: Predicted targets for unclassified non-conserved miRNAs in radish.

miRNA family	Target sequence	Gene ID	Target gene	Target gene annotation
miR1077	Unigene6704	AT4G22780	*ACR7*	ACT domain repeat 7 protein
miR1172	CL1185.Contig1	AT4G13930	*SHM4*	serine hydroxymethyltransferase 4
	Unigene17878	AT4G38440		transcriptional elongation regulator MINIYO
	Rsa#S42022008	AT5G09860	*THO1*	THO complex subunit 1
miR1220	CL3299.Contig1	AT4G37300	*MEE59*	protein maternal effect embryo arrest 59
miR1432	Unigene18163	AT3G60320		hypothetical protein
	Unigene18165	AT3G60320		hypothetical protein
	Rsa#S42021073	AT5G62390	*BAG7*	BCL-2-associated athanogene 7
miR1510	CL7101.Contig2	AT1G27900		RNA helicase family protein
	CL7101.Contig3	AT1G27900		RNA helicase family protein
miR1535	Unigene5301	AT2G26460	*SMU2*	RNA splicing protein SMU2
	Unigene20428	AT1G14520	*MIOX1*	inositol oxygenase 1
miR1846	Unigene4941	AT1G36320		hypothetical protein
miR1847	Unigene668	AT5G51350		receptor-like kinase MOL1
miR1858	Unigene34	Arthcp087	*ycf1*	ycf1
	Unigene18893	Arthcp087	*ycf1*	ycf1
	Rsa#S42005929	AT4G04770	*ABC1*	ATP binding cassette protein 1
miR1871	Unigene16275	AT1G21280		hypothetical protein
miR2092	Unigene7810	AT2G25490	*EBF1*	EIN3-binding F-box protein 1
miR3437	Unigene13529	AT2G29660		hypothetical protein
miR3513	CL1280.Contig3	AT3G51850	*CPK13*	calcium-dependent protein kinase 13
	Rsa#S42024820	AT4G33300	*ADR1-L1*	putative disease resistance protein ADR1-like 1
	Rsa#S42031847	AT3G51850	*CPK13*	calcium-dependent protein kinase 13

miR4395	Rsa#S41987342	AT2G18328	*RL4*	protein RADIALIS-like 4
miR5054	Unigene7893	AT2G24580		putative sarcosine oxidase
	Rsa#S42568393	AT4G38100		protein CURVATURE THYLAKOID 1D
miR5077	CL3983.Contig1	AT3G59970	*MTHFR1*	methylenetetrahydrofolate reductase 1
	CL3983.Contig2	AT3G59970	*MTHFR1*	methylenetetrahydrofolate reductase 1
	CL3983.Contig3	AT3G59970	*MTHFR1*	methylenetetrahydrofolate reductase 1
	CL3983.Contig4	AT3G59970	*MTHFR1*	methylenetetrahydrofolate reductase 1
	Rsa#S42586689	AT3G59970	*MTHFR1*	methylenetetrahydrofolate reductase 1
miR5083	CL4117.Contig1	AT5G44530		Subtilase family protein
	CL4117.Contig2	AT5G44530		Subtilase family protein
miR5137	Unigene12524	AT3G61800		hypothetical protein
	Unigene19750	AT3G60300		RWD domain-containing protein
miR5139	CL74.Contig4	AT5G47490		RGPR-related protein
	CL8918.Contig2	AT5G67460		O-Glycosyl hydrolases family 17 protein
	Unigene23324	AT2G41970		putative protein kinase
	Rsa#S41993046	AT1G34070		hypothetical protein
	FD956528	AT3G48140		B12D protein
miR5368	CL2178.Contig1	AT1G72040		deoxyribonucleoside kinase
miR5490	CL9355.Contig1	AT1G71960	*ABCG25*	ABC transporter G family member 25
	Rsa#S41991342	AT3G54050	*HCEF1*	fructose-1,6-bisphosphatase
miR5501	CL2187.Contig1	AT1G75730		hypothetical protein
	CL2187.Contig2	AT1G75730		hypothetical protein
	Unigene9681	AT5G23110		hypothetical protein
miR5562	CL7101.Contig3	AT1G27900		RNA helicase family protein
	Unigene7364	AT3G18370	*ATSYTF*	protein ATSYTF
miR5649	CL6112.Contig2	AT4G14150	*PAKRP1*	phragmoplast-associated kinesin-related protein 1

miR5665	CL1233.Contig1	AT4G35230	*BSK1*	BR-signaling kinase 1
miR5670	CL3895.Contig1	AT5G25140	*CYP71B13*	cytochrome P450 71B13
miR5671	CL662.Contig1	AT1G63440	*HMA5*	putative copper-transporting ATPase HMA5
	CL662.Contig2	AT1G63440	*HMA5*	putative copper-transporting ATPase HMA5
	CL3190.Contig2	AT3G26710	*CCB1*	cofactor assembly of complex C
	CL3190.Contig3	AT3G26710	*CCB1*	cofactor assembly of complex C
	Rsa#S43009223	AT3G26710	*CCB1*	cofactor assembly of complex C
	FD956099	AT5G29000		protein PHR1-LIKE 1
miR5672	CL7101.Contig2	AT1G27900		RNA helicase family protein
	CL7101.Contig3	AT1G27900		RNA helicase family protein
miR5751	Unigene14038	AT3G16270		ENTH/VHS family protein
	Unigene15953	AT4G33080		AGC (cAMP-dependent, cGMP-dependent and protein kinase C) kinase family protein
	Rsa#S42007531	AT2G18100		hypothetical protein
miR576	CL2364.Contig1	AT5G27520	*PNC2*	peroxisomal adenine nucleotide carrier 2
	CL9249.Contig1	AT3G54300	*VAMP727*	vesicle-associated membrane protein 727
	Unigene16216	AT5G54910		DEAD-box ATP-dependent RNA helicase 32
	Unigene28076	AT4G01640		F-box associated ubiquitination effector family protein
miR6144	CL2750.Contig1	AT2G29980	*FAD3*	omega-3 fatty acid desaturase
miR6170	CL2072.Contig1	AT3G56140		hypothetical protein
	CL2072.Contig2	AT3G56140		hypothetical protein
	FD956117	AT2G03870		U6 snRNA-associated Sm-like protein LSm7
miR6180	CL4200.Contig1	AT4G08350	*GTA2*	global transcription factor group A2
	CL4645.Contig2	AT2G45990		hypothetical protein
	CL7004.Contig2	AT5G16370	*AAE5*	acyl activating enzyme 5
	Unigene9128	AT4G11800		calcineurin-like phosphoesterase domain-containing protein

	Unigene12080	AT1G60190		U-box domain-containing protein 19
	Unigene22802	AT3G13040		myb-like HTH transcriptional regulator family protein
miR6184	Unigene8323	AT4G09490		polynucleotidyl transferase, ribonuclease H-like superfamily protein
miR6196	Rsa#S41976403	AT5G64460		phosphoglycerate mutase-like protein
miR6267	Unigene26067	AT3G55150	*EXO70H1*	exocyst subunit exo70 family protein H1
miR6300	Unigene7892	AT4G34980	*SLP2*	subtilisin-like serine protease 2
miR6441	CL795.Contig4	AT5G23610		hypothetical protein
	CL795.Contig5	AT5G04840		bZIP protein
	CL7101.Contig2	AT1G27900		RNA helicase family protein
miR6454	CL1127.Contig2	AT4G36970		Remorin family protein
	CL1127.Contig3	AT4G36970		Remorin family protein
	CL7101.Contig3	AT1G27900		RNA helicase family protein
	Rsa#S42018777	AT1G32370	*TOM2B*	tobamovirus multiplication 2B protein
	Rsa#S42043371	AT4G23880		hypothetical protein
miR6471	CL41.Contig1	AT2G01021		hypothetical protein
	Unigene2870	AT3G27960		tetratricopeptide repeat domain-containing protein
miR6483	CL6432.Contig1	AT4G22180		putative F-box protein
miR7126	Unigene20370	AT1G07860		putative protein kinase
	Unigene21704	AT3G21740	*APO4*	APO protein 4
miR7510	Unigene15577	AT5G59550		ABA- and drought-induced RING-DUF1117 protein
miR7696	Unigene13146	AT2G17480	*MLO8*	MLO-like protein 8
	Unigene19469	AT5G50790		bidirectional sugar transporter SWEET10
	Rsa#S42034008	AT1G21200		sequence-specific DNA binding transcription factor
	Rsa#S43008405	AT5G50790		bidirectional sugar transporter SWEET10
miR7698	CL3003.Contig2	AT1G18840	*IQD30*	protein IQ-domain 30
	CL9922.Contig2	AT5G18370		TIR-NBS-LRR class disease resistance protein

	Unigene18994	AT5G38344		Toll-Interleukin-Resistance domain-containing protein
miR7767	CL128.Contig4	AT1G10390		nucleoporin autopeptidase
	CL2610.Contig3	AT1G80640		probable receptor-like protein kinase
	CL7445.Contig1	AT1G64330		myosin heavy chain-related protein
	CL9385.Contig1	AT1G64330		myosin heavy chain-related protein
	CL9385.Contig2	AT1G20640		nodule inception protein-like protein 4
	CL9387.Contig1	AT1G13790		SGS3-like protein FDM4
	CL9731.Contig2	AT1G60550	*ECHID*	1,4-Dihydroxy-2-naphthoyl-CoA synthase
	Unigene697	AT5G63190		MA3 domain-containing protein
	Unigene5787	AT3G07270		GTP cyclohydrolase I
	Unigene9558	AT1G31360	*RECQL2*	mediator of RNA polymerase II transcription subunit 34
	Unigene10044	AT3G19150	*KRP6*	cyclin-dependent kinase inhibitor 6
	Unigene10082	AT2G47440		tetratricopeptide repeat-containing protein
	Unigene15909	AT4G24220	*VEP1*	3-oxo-Delta(4,5)-steroid 5-beta-reductase
	Rsa#S42024665	AT4G40060	*HB16*	homeobox-leucine zipper protein ATHB-16
	Rsa#S42026112	AT1G64330		myosin heavy chain-related protein
	Rsa#S42016265	AT5G44820		Nucleotide-diphospho-sugar transferase family protein
	Rsa#S42021405	AT5G44820		Nucleotide-diphospho-sugar transferase family protein
	Rsa#S42568034	AT5G63640		ENTH/VHS/GAT family protein
	Rsa#S43005762	AT3G48390		MA3 domain-containing protein
	Rsa#S43026869	AT3G18295		hypothetical protein
	FD955301	AT3G18295		hypothetical protein
miR8030	Rsa#S43024877	AT1G29690	*CAD1*	protein constitutively activated cell death 1
	FD951475	AT3G55530	*SDIR1*	E3 ubiquitin-protein ligase SDIR1
miR812	Rsa#S42006970	AT1G66850		bifunctional inhibitor/lipid-transfer protein/seed storage 2S albumin-like protein
miR831	CL7101.Contig3	AT1G27900		RNA helicase family protein

miR845	Unigene17362	AT2G45740	*PEX11D*	peroxisomal membrane protein 11D
miR852	CL10031.Contig2	AT4G32040	*KNAT5*	homeobox protein knotted-1-like 5
miR858	CL3556.Contig1	AT3G26360		ribosomal protein S21 family protein
	Rsa#S42018117	AT2G31980	*CYS2*	cysteine proteinase inhibitor 2
	FD957240	AT2G31980	*CYS2*	cysteine proteinase inhibitor 2
	Rsa#S42048096	AT5G02160		hypothetical protein
miR894	CL3983.Contig1	AT3G59970	*MTHFR1*	methylenetetrahydrofolate reductase 1
	Rsa#S42586689	AT3G59970	*MTHFR1*	methylenetetrahydrofolate reductase 1
miR952b	Unigene21106	AT5G49950		alpha/beta-Hydrolases superfamily protein
